# Synthesis and Characterization of Poly (β-amino Ester) and Applied PEGylated and Non-PEGylated Poly (β-amino ester)/Plasmid DNA Nanoparticles for Efficient Gene Delivery

**DOI:** 10.3389/fphar.2022.854859

**Published:** 2022-04-08

**Authors:** Sajid Iqbal, Alessandro F. Martins, Muhammad Sohail, Jingjing Zhao, Qi Deng, Muhan Li, Zhongxi Zhao

**Affiliations:** ^1^ Department of Pharmaceutics, Key Laboratory of Chemical Biology of Ministry of Education, School of Pharmaceutical Sciences, Cheeloo College of Medicine, Shandong University, Jinan, China; ^2^ Laboratory of Materials, Macromolecules, and Composites (LaMMAC), Federal University of Technology - Paraná (UTFPR), Apucarana, Brazil; ^3^ Group of Polymers and Composite Materials (GMPC), Department of Chemistry, State University of Maringá (UEM), Maringá, Brazil; ^4^ Department of Chemical and Biological Engineering, Colorado State University (CSU), Fort Collins, CO, United States; ^5^ Key Laboratory of Molecular Pharmacology and Drug Evaluation, Yantai University, Yantai, China; ^6^ Key University Laboratory of Pharmaceutics and Drug Delivery Systems of Shandong Province, School of Pharmaceutical Sciences, Cheeloo College of Medicine, Shandong University, Jinan, China; ^7^ Pediatric Pharmaceutical Engineering Laboratory of Shandong Province, Shandong Dyne Marine Biopharmaceutical Company Limited, Rongcheng, China; ^8^ Chemical Immunopharmaceutical Engineering Laboratory of Shandong Province, Shandong Xili Pharmaceutical Company Limited, Heze, China

**Keywords:** poly (β-amino ester), biodegradable polymer, stable polyplexes, gene delivery, transfection

## Abstract

Polymer-based nanocarriers require extensive knowledge of their chemistries to learn functionalization strategies and understand the nature of interactions that they establish with biological entities. In this research, the poly (β-amino ester) (PβAE-447) was synthesized and characterized, aimed to identify the influence of some key parameters in the formulation process. Initially; PβAE-447 was characterized for aqueous solubility, swelling capacity, proton buffering ability, and cytotoxicity study before nanoparticles formulation. Interestingly, the polymer-supported higher cell viability than the Polyethylenimine (PEI) at 100 μg/ml. PβAE-447 complexed with GFP encoded plasmid DNA (pGFP) generated nanocarriers of 184 nm hydrodynamic radius (+7.42 mV Zeta potential) for cell transfection. Transfection assays performed with PEGylated and lyophilized PβAE-447/pDNA complexes on HEK-293, BEAS-2B, and A549 cell lines showed better transfection than PEI. The outcomes toward A549 cells (above 66%) showed the highest transfection efficiency compared to the other cell lines. Altogether, these results suggested that characterizing physicochemical properties pave the way to design a new generation of PβAE-447 for gene delivery.

## Graphical Abstract

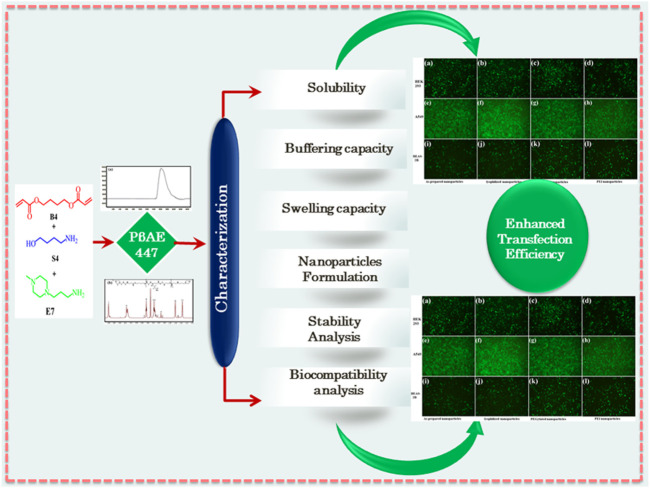

## Introduction

The rational designing of a vector is essential for the programmed transport of cargo to desired sites. In the academic field, various drug delivery systems (DDSs) have been abundantly introduced. Yet, these nanoplatforms have not achieved efficient *in vivo* gene expressions (Gonçalves and Paiva 2017). However, gene therapy is the only option to cure underlying genetic defects rather than managing symptoms. As of November 2020, 1,645 clinical trials out of 4,500 have been completed and 545 were in phase 3/4. This shows that gene therapy is developing rapidly and gradually translating into clinical practice. In all these trials, the lack of efficient and safe nanocarrier remains the most critical bottleneck (Yu et al., 2021). Although viral vectors are frequently used, non-viral vectors are also becoming more common.

In the domain of non-viral vectors, research on cationic polymers has been expanded since 2004. Substantial synthesis has developed advanced polymeric vectors with excellent cellular uptake toward cells and sustainable buffering capacities. Chemical modifications have improved the bioavailability of therapeutic materials at desired sites (Zeng et al., 2017). Besides, cationic polymers also receive attention because they stabilize negatively charged nucleic acids at physiological pH, forming polymer/gene nanocomplexes. This association protects plasmid DNA (pDNA) from enzymes, enhancing cargo-carrying capacities and promoting predefined unpacking payloads by supporting the controlled pDNA release (Chen et al., 2020). However, most of the cationic polymers are non-degradable, may accumulate in tissues particularly after continuous administration. Cytotoxicity of these polymers is another major issue generated from the loss of cytoplasmic proteins due to adverse interactions with membranes (Zou et al., 2009). Therefore, there is a persistent need to develop a biodegradable gene carrier, both in terms of payload capacity and capsid engineering.

Poly (β-amino ester)s (PβAEs) are the key family members of cationic synthetic and biodegradable polymers. They present tunable structures and potentialities for chemical functionalization (Iqbal and Zhao 2021). The structural modifications on PβAEs have resulted in stable and small size nanoparticles with pDNA, miRNA, and siRNA with target capacities toward tumor cells (Kim et al., 2020). The ester, amino, and disulfide moieties in their chemical structures resemble the chemical structures of glycosaminoglycans, proteoglycans, and proteins found on extracellular membrane matrices (Iqbal et al., 2020). PβAEs offer flexible design chemistries because they can be prepared from different monomers via Michael’s addition, generating a vast combinatorial library with various kinetics profiles for different delivery purposes (Li et al., 2013; Rui et al., 2017). Until now, more than 2,350 PβAEs have been synthesized and evaluated for gene transfections in various cell lines (Wang et al., 2020). However, all of these PβAEs are not suitable gene carriers and much characterization is needed before use (Xia et al., 2011). A significant exception to this trend is the poly (β-amino ester)-447 (PβAE-447). It is more effective for gene delivery (Wilson et al., 2018) because the monomer 1,4-butanediol diacrylate (“B4”) and the end-capping group 1-(3-aminopropyl)-4-methyl piperazine (“E7”) have demonstrated high capacities for cell internalization (Shmueli et al., 2012; Kim et al., 2014). Although all these features make PβAE-447 an attractive pDNA delivery vector, it should be noticed that simple PβAE-447 nanoparticles are susceptible to aqueous degradation (Wilson et al., 2019). This limitation is more pronounced especially in the case when the final PβAE-447 and their nanoparticles are stored for long-term studies. Besides; the nanoparticle features; including size, charge density, and other physicochemical properties such as stability and particles aggregation have considerable influence on nanoparticles bioactivity (Zeb et al., 2020). Therefore, precise characterization of the polymer is necessary before its application (Zhang et al., 2016). Several studies have been conducted to optimize PβAEs architecture (Green et al., 2008) to formulate nanoparticles with desired characteristics for gene delivery (Perni and Prokopovich, 2020). However, reported studies have focused on a single variable such as slow-release (Helaly and Hashem, 2013). While in reality, PβAE-based nanocomplexes face a broad spectrum of bio-physicochemical challenges, including colloidal stability, swelling response, and incubation time.

For the first time, only PβAE-447 was characterized to improve its reproducibility and efficacy for enhanced cell transfection. In this regard, several different assays (solubility, stability, and buffering properties) were performed to analyze the critical formulation determinants that could affect the PβAE-447 mediated gene delivery. The surface was functionalized with polyethylene glycol (PEG) to improve colloidal stability. Besides, the incubation time for efficient cellular uptake was also explored. These approaches can be used to discover the parameter ranges that would produce optimized transfections and ensure robust reproducibility.

## Materials and Methods

### Materials

The 1-(3-aminopropyl)-4-methyl piperazine (E7), 1,4-butanediol diacrylate (B4), 4-amino-1-butanol (S4), and Polyethylenimine (PEI) were obtained from Alfa Aesar (Beijing, China). Dimethyl sulfoxide (DMSO), N-hexane, dimethylformamide (DMF), Ethyl acetate (EtOAc), Dichloromethane (DCM), tetrahydrofuran (THF), and diethyl ether were purchased from Sigma-Aldrich (Beijing, China). Methoxy Polyethylene glycol-succinimidyl succinate (mPEG-SS) commonly referred to as PEG of 5 kDa was acquired from Shanghai Titan Technology Co., Ltd (Shanghai, China).

The cell culture media and all other reagents were used as received. Aqueous solutions of sodium acetate (NaAc, pH 5.1 ± 0.1 at 0.025 M), sodium chloride (NaCl, 150 mM), and sodium hydroxide (NaOH, 0.1 N) were prepared and sterilized. Plasmid DNA (pDNA) expressing green fluorescent protein (GFP) was prepared accordingly, while growth media and Hoechst dye were stored according to the manufacturer’s instructions.

### Poly (β-amino ester)-447 Synthesis

**FIGURE 1:**
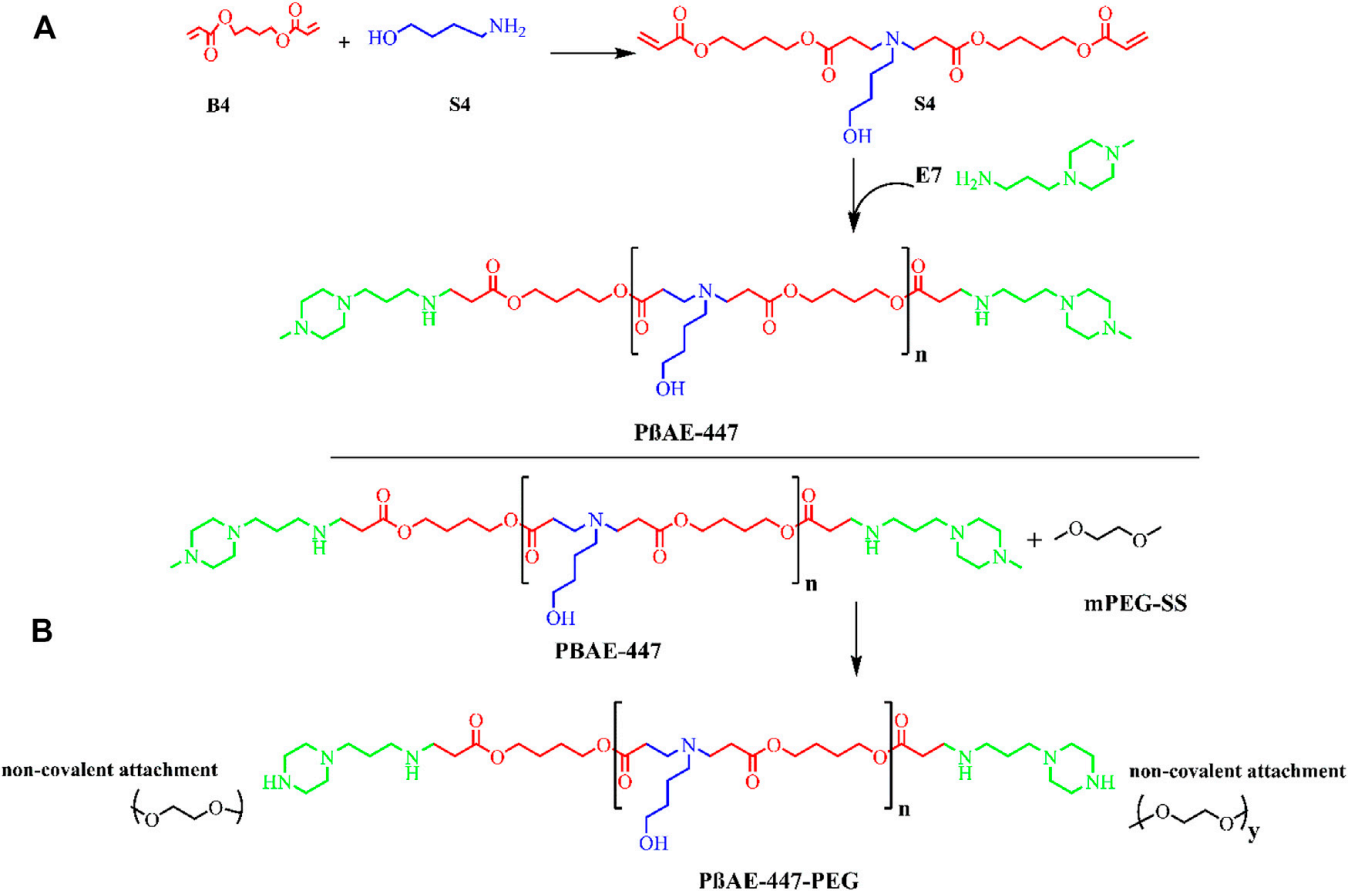
Schematic synthesis reaction mechanism: **(A)** poly (β-amino ester)-447 (PβAE-447) synthesis **(B)** PβAE-447-PEG.

Poly (β-amino ester)-447 (PβAE-447) was synthesized following a previously published procedure (Smith et al., 2017), with alterations as outlined in (Figure 1). In the first step, the monomers B4 and S4 were mixed at a 1:1 M ratio and stirred (1,000 rpm) overnight at 90°C to yield the polymer B4-S4 (Figure 1).

In the second step, the polymer B4-S4 was dissolved in THF at 100 mg/ml and then combined with E7 (polymer end-capping group) in THF (0.2 M) at 500 rpm for 2 hours at room temperature. The PβAE-447 was precipitated in cold diethyl ether and washed to remove the residual monomers. The polymer was dispersed again in diethyl ether to guarantee the removal of the remaining monomers. The polymer was vacuumed dried for 2 days to remove traces of solvents. The dry and clean polymer was dissolved in DMSO at 100 mg/ml and stored in small aliquots at −20 °C for further use.

The PβAE-447 was PEGylated. For this, PEG (2.05 M equivalent) and the prepared PβAE-447 solution were transferred into a glass vial, vacuumed, and then purged with nitrogen. The mixture was then reacted in anhydrous THF at room temperature overnight. The PEGylated PβAE was washed two times in cold diethyl ether, and then vacuumed dried. The PEGylated PβAE-447 was separately dissolved in DMSO at 100 mg/ml and stored at −20°C for further use.

### Characterization

The polydispersity and molecular weight (M_w_) of the PβAE-447 were determined by gel permeation chromatography (GPC). The polymer stock solution in DMSO was diluted in THF (100%) at 5.5 mg/ml. The prepared polymer solution was filtered in a 0.2 μm polytetrafluoroethylene syringe filter before flowing through a waters 515 liquid chromatograph equipped with three styragel columns and 2,414 refractive index detector at a flow rate of 1.0 ml/min at 40°C and then analyzed with Breeze two software. The number average molecular weight (M_n_) and M_w_ were determined using the polystyrene standard.

The purified and dried PβAE-447 was dissolved in deuterated chloroform (CDCl_3_) at 10 mg/ml and then analyzed by ^1^H NMR spectroscopy, using a Bruker instrument 400 MHz, Topspin 2.0 (Toronto, Canada).

### Polymer Solubility

Different aliquots of the PβAE-447 were mixed with NaAc buffer (25 mM), forming polymer suspensions (“milky” appearance). After sonication (15 min), the samples were kept in an orbital shaker at room temperature for 1 h. Exemplary, 1, 3, 5, 7, 10, and 20 mg of PβAE-447 were dissolved in NaAc (1 ml), and four wells of 96-well plate were used for each concentration. The absorbance of the systems (each well) was recorded with a plate reader at 620 nm. The polymer solubility was confirmed with the naked eye as well as by plotting the recorded absorbance at 620 nm. For this, the absorbance of the polymer systems was compared with the absorbance of references (NaAc and DMSO used to prepare the polymer systems) (Sunshine et al., 2011).

### Acid-Base Titration

An acid-base titration was performed to evaluate the buffering capacity of PβAE-447. The polymer from its stock solution in DMSO (100 mg/ml) was diluted to 1 mg/ml in NaCl (2.0 ml, 150 mM), and then NaOH (0.1 M) was used to adjust the pH to 10. The pH of the solution (2.0 ml) was reduced to 3 with HCl (0.1 N). The pH alteration was continuously monitored (Gong et al., 2018). Distilled water was titrated in the same way to compare the pH changes as the polymer was titrated. The pH values of the polymer solution were recorded each time after the repeated addition of HCl. A pH meter (pH 211 microprocessor pH meter, HANA Instruments, Seoul, South Korea) was used to measure the pH constantly. The slope of the line in the plot for pH and the concentration of HCl used show the intrinsic buffering ability. The proton buffering capacity of the polymer was calculated through

$$\text{Buffering capacity}=\frac{\Delta {\text{V}}_{\text{HCl}}\;\times {\text{ C}}_{\text{HCl}}}{\text{m}}$$ (1)

where ΔV_HCl_ is the HCl volume, C_HCl_ is the concentration of HCl, and m is the polymer mass (Hwang et al., 2014).

### Swelling Capacity

The swelling property of the PβAE-447 was evaluated by immersing dried polymer disks (0.1 g) in DMSO, THF, DCM, and EtOAc at room temperature for a defined time. The dried mass for each polymer disk was called W_o_, and the swollen polymer disk mass was labeled W_s_. The polymer disks were taken out from the solvents at calculated times. The solvents in excess on the disks were gently removed with filter papers before measuring W_s_ (Biswal et al., 2011). The swelling degree (SD) was determined at different times after contact with the solvents. The SD (%) measurements were determined through .

$$SD=\frac{W_{S}-W_{0}}{\;W_{0}}\;\times \;100$$ (2)

### Cytotoxicity Assay

The relative cytotoxicity of pure and PEGylated PβAE-447 was separately investigated by using the 3-(4,5-dimethylthiozol-2-yl)-2,5-diphenyl tetrazolium bromide (MTT) assay. Human embryonic kidney (HEK-293), bronchial epithelial (BEAS-2B), and lung adenocarcinoma epithelial (A549) cells were incubated in 96-well plates with DMEM (100 μL) for 24 h. When the cells achieved almost 70% confluence, the old media was removed. The new media containing various concentrations of pure and PEGylated PβAE-447 were added to each well. The 96 well plates were incubated for 24 h, and then 25 μL MTT solution (5.0 mg/ml) in PBS was added to each well. After 4 h of incubation, the MTT solution and DMEM were aspirated, and 150 μL DMSO was added to dissolve the formazan crystals. The plates were placed on a shaker for 10 min before recording the absorbance at 570 nm by an ELISA microplate reader (Bio-Rad, California, United States). The percentage of cell viability was calculated by using Eq. (3)

$$\text{Cell viability }\left(\text{\%}\right)=\left(\frac{{\text{A}}_{\text{sample}}}{{\text{A}}_{\text{control}}}\right)\;\times \;100$$ (3)

where A_sample_ is the absorbance from the treated cells and A_contro_l is the absorbance from untreated cells.

Moreover, the cell viability of PEI 25 KDa was also determined.

### Nanoparticles Formulation and Characterization

The pDNA and PβAE-447 were diluted to 0.06 and 3.6 μg/ml in NaAc (25 mM, pH 5.0) at room temperature, respectively. The polymer concentration was 60-fold higher than the pDNA concentration (PβAE-447/pDNA weight ratio equal to 60).

For preparing the PβAE-447/pDNA nanocomplexes, the diluted polymer (50 µL) was added to 50 µL pDNA and gently mixed by pipetting. The prepared suspension was kept for 10 min under rest to facilitate the self-assembling of the nanocomplexes. The as-prepared nanoparticle suspension was characterized by dynamic light scattering (DLS). Hydrodynamic radius and Zeta potential measurements were determined in a Malvern Zetasizer Nano (ZS) instrument (NanoSight Ltd, United Kingdom) at room temperature (Kamat et al., 2013).

An as-prepared nanoparticle suspension was mixed with sucrose (30 mg/ml in 25 mM NaAc), performing a 1:1 v/v nanoparticle/sucrose mixture to assess the nanoparticles’ stability over time. The mixture was frozen for 2 h at −80°C, lyophilized for 2 days, and stored at 4°C. The lyophilized nanoparticles were re-dispersed in water (30 mg/ml) for further DLS analysis (Zeta potential and hydrodynamic radius). These nanoparticles were called lyophilized nanoparticles.

### Nanoparticle Stability Analysis

The stability of the PβAE-447/pDNA and PEGylated PβAE-447/pDNA nanoparticles was evaluated in fetal bovine serum (FBS) and NaCl. The polyplexes were prepared the same way as mentioned above. Then FBS (10% w/v) and NaCl (300 mmol) were separately added to the nanoparticle’s suspensions. The size and Zeta potential of the resulting suspensions were measured after 4 h of incubation at room temperature (Zeng et al., 2017).

### Nanoparticles Hemagglutination Study

The agglutinating activity of PβAE-447/pDNA formulated at 60 wt/wt was examined in a 96-well plate. Briefly, fresh mice blood was centrifuged at 1000 rpm for 15 minutes. The supernatant containing plasma and buffy coat were removed. The erythrocytes were washed three times with PBS. After each cycle, the supernatant was carefully discarded. The red blood cells obtained in this method were found to be pure from cell debris and leucocytes. The erythrocytes were diluted in PBS to a final concentration of 2 % (v/v). 50 μL from this dilution was transferred to a 96-well microplate. The PβAE-447/pDNA complexes were added to the RBC suspension (1:1) and incubated for 30 minutes at room temperature. After incubation, hemagglutination was observed with the naked eye as well as under an optical microscope. The experiment was performed in triplicate.

### Cell Transfection

HEK-293, BEAS-2B, and A549 cells) cell lines were grown at a density of 12,500 cells/well in 100 μL media in three separate 96-well plates for 24 h to allow cell adhesion. pDNA was dissolved in NaAc (25 mM, pH 5.0) to 0.06 μg/μL. Polymer (stock solution in DMSO) was diluted in NaAc (25 mM, pH 5.0) to 3.6 μg/μL (60 polymer/p-DNA weight ratio). The diluted pDNA (30 μL) and diluted polymer (30 μL) solutions were mixed for 5 seconds with a vortex mixer. The mixture was kept at rest for 10 minutes to promote the formation of the nanoparticles. Then, 20 μL nanoparticle suspension was added to 100 μL of the cell growth media.

Before adding the polymer/pDNA nanoparticles (as-prepared, lyophilized, and pegylated PβAE-447/pDNA nanoparticles) over the seeded cells, the media was aspirated, and new media containing mentioned nanoparticles were used for transfection. After 4 h of incubation, the media containing nanoparticles was poured out from each well, and an equal volume of fresh and prewarmed media was added. The 96-wells plates were kept in an incubator at 37°C under 5% CO_2_ and examined for cell transfection after 48 h.

Control experiments with PEI (MW 25,000) were also performed as above. PEI/pDNA complexes were formulated at a 3:1 PEI to pDNA weight ratio in NaCl (25 mM, pH 5.0). PEI/pDNA suspension was shaken vigorously and then incubated for 15 min at room temperature. PEI/pDNA was added to cells for the final concentration of pDNA 600 ng/well.

A semi-quantitative analysis was performed on the images captured by fluorescent microscope (Carl Zeiss MicroImaging GmbH, Germany) to calculate the mean fluorescent intensity of GFP-expressed cells using the ImageJ (http://rsb.info.nih.gov/ij).

### Confocal Microscopy

Four different experiments were performed to investigate the effect of the incubation period of the PEGYlated PβAE-447/pDNA nanoparticles on cellular uptake.

The different cell lines (HEK-293, BEAS-2B, and A549) were cultured in 96-wells for 24 h for cell adhesion. Nanocomplexes in culture media were added to the attached cells for different time periods. After the predefined incubation time intervals, the media containing the nanoparticles were replaced by new media, and the plates were incubated for 48 h. Afterward, the culture media was removed, and the cells were incubated for 10 min in the Hoechst dye 33,342 solutions. Immediately before confocal imaging, the media from the wells were aspirated, and the cells were rinsed twice with PBS(1X). Confocal microscopy images were recorded with a confocal laser scanning microscopy (GmbH Wetzlar, Germany).

### Analysis

All the experiments were performed in triplicate (*n* = 3), and the results were expressed as the mean ± standard deviations.

## Result

### Synthesis and Characterization of the Poly (β-amino Ester)

In this study, PβAE-447 was synthesized following the Michael addition reaction mechanism by polymerizing 1,4-butanediol diacrylate “B4” and 4-amino-1-butanol “S4” stoichiometrically. The reaction formed a polymer base that was end-capped with 1-(3-aminopropyl)-4-methyl piperazine (E7). The yielded polymer obtained from the monomers “B4”, “S4”, and the end-capping reagent “E7” is named as 1-(3- aminopropyl)-4-methyl piperazine end-modified poly (1,4-butanediol diacrylate-*co*-4-amino-1- butanol) (PβAE-447). This nomenclature depends on four carbon atoms between the acrylate groups in “B4” and four carbon atoms across the amine and the alcohol groups in” S4” (Li et al., 2013). The polymerization of PβAEs can be carried out in a wide range of solvents. However, DMSO was preferred in the synthesis because it is a commonly used solvent in bio-assays including cellular-based assays.

**FIGURE 2:**
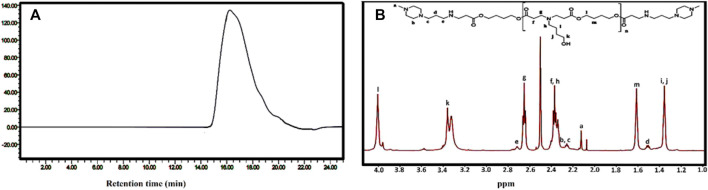
Characterization of PβAE-447: **(A)** GPC chromatogram and **(B)** ^1^H NMR spectrum in CDCl_3_.

The PβAE-447 was characterized by gel permeation chromatography (GPC) to determine the molecular weight and polydispersity index (PDI) (Figure 2A). The GPC chromatogram shows the presence of a single polymer with Mn of 5,354, Mw of 9,575, MP of 4,934, and PDI of 1.7.

The chemical structure of PβAE-447 was analyzed by ^1^H NMR (Figure 2B), and the spectrum matches with previously published results (Chemical shift analysis Supplementary Figure S2). The PβAE-447 ^1^H NMR spectrum shows peaks assigned to hydrogen atoms found on the monomers B4 and S4 and the disappearance of the acrylate peaks at the end of both sides of ppm shows completion of the end-capping reaction.

### Solubility

The well-established method for investigating the carrier solubility is adding an extra quantity of carriers to a fixed buffer volume at a set pH. A 96-well plate absorbance assay was used to quantitatively evaluate the PβAE-447 solubility at 620 nm. The data (not shown) shows that PβAE-447 was completely soluble at 10 μg/μL in a NaAc buffer. The gene release depends on the PβAE-447 solubility inside the cells. The water-soluble and biodegradable cationic polymer has hydrolyzable ester bonds in its backbone (Agarwal et al., 2012). The polymer degradation should support the release of genetic materials in an active form, resulting in better gene expression. Other strategies can also be used to modify the hydrophilic PβAE nature, including its *N*-quaternization and pegylation. These processes should increase the aqueous solubility and enhance pDNA release at physiological pH.

### Proton-Buffering Capacity

**FIGURE 3:**
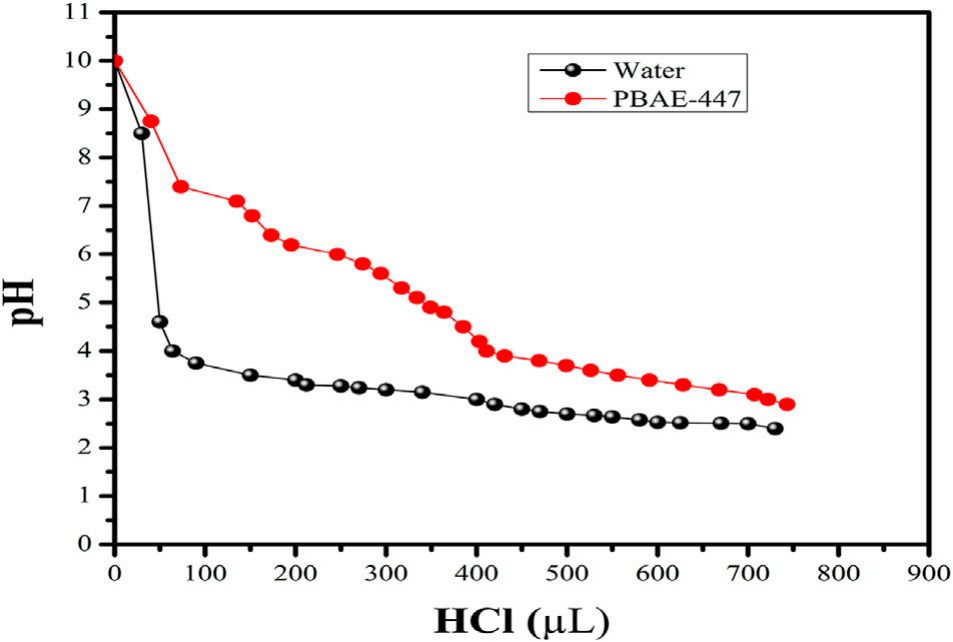
Buffering capacity of the PβAE-447 is determined by titration.

For evaluating the buffering capacity of PβAE, direct polymer titration with HCl was performed. For comparison, the polymer titration was repeated with distilled water. It is pretty clear from (Figure 3) that approximately 0.013 mmol HCl is needed to change the pH from 7.4 to 5.1 (*i.e*., endosomal pH range). PβAEs with the end-capping group E7 have high buffering capacities because the E7 comprises two tertiary amines. Similarly, PβAEs with the end-capping E6 would have high buffering capabilities than E4 and E6 groups. It has been hypothesized that the nanoparticles remain in the endosomes if the carriers have low buffering capacities (Sunshine et al., 2012). It suggests no relationship between cell uptake and buffering capacity. Still, there is a strong relationship between transfection efficiency and buffering capacity.

Additionally, the nanoparticles formulated at a 75 w/w PβAE-447/pDNA ratio would have a higher buffering capacity than at 60 w/w, because of the high polymer content at 75 w/w. The PβAE-447 can have different proton buffering capacities depending on its concentration. Moreover, the monomer ratio used to synthesize the PβAE-447 could also influence the proton buffering capacity because the amino group content in the polymer structure depends on the S4 concentration.

The titration curve for the water decreased promptly in the pH range between 4 and 10, and the PβAE-447 curve displayed a delayed pH reduction, suggesting that PβAE has a high buffering capability (Figure 3). Thus, the buffer capacity should accelerate endosomal escape and enhance the transfection toward treated cells that have already taken up the particles.

### Swelling Studies

**FIGURE 4:**
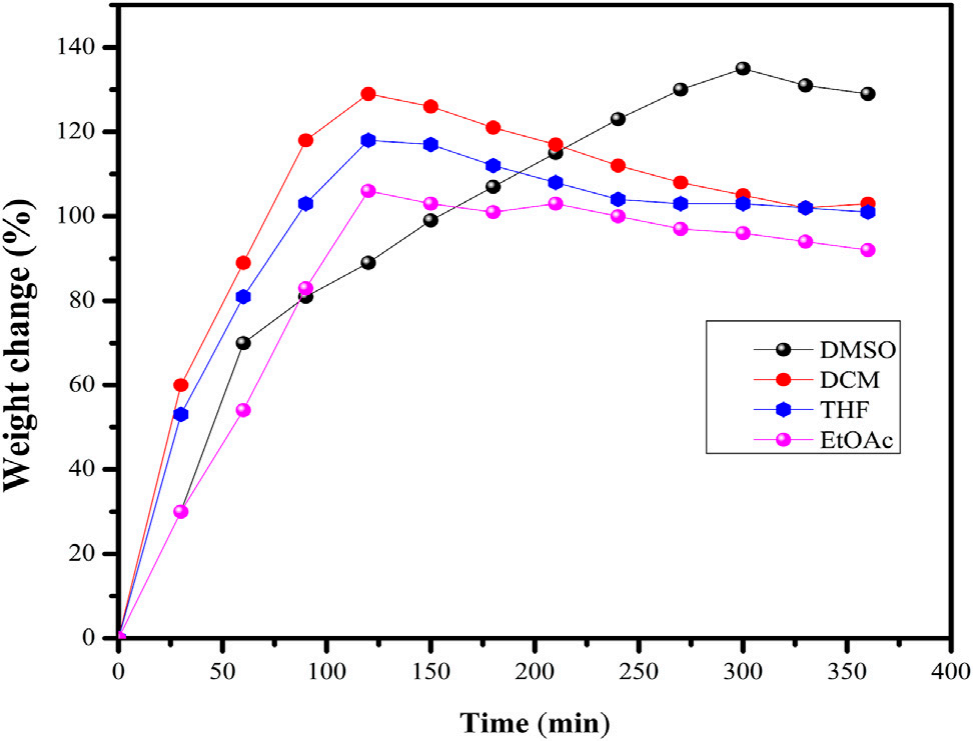
The swelling degree (SD%) of the PβAE-447 in different solvents.

The PβAE-447 SD was investigated in conventional solvents such as DMSO, DCM, THF, and EtOAc at room temperature for 0, 100, 250, and 360 min (Figure 4). The PβAE-447 swelling increases as time rises, achieving the equilibrium condition. The maximum swelling is reached in approximately 2 h in DCM, THF, and EtOAc, whereas in DMSO, the maximum swelling occurs in around 5 h. The polymer SD reduces in the followed order DMSO > DCM > THF > EtOAc. The swelling is rationalized with the solvent-polymer interaction theory that predicts polymer solubility (Biswal et al., 2011). Solvent polymer interactions have a critical role in polymer synthesis. Maximized polymer-solvent interactions may lead to chain expansion, which is important to optimize material’s processing; for example, resultant mechanical properties (Ferrell et al., 2017). PβAE-447 demonstrated an ability to absorb conventional solvents and increased in size, as revealed by the swelling behavior stated in Figure 4. The PβAE-447 could have a transition in the size through solvents absorption as a stimulus which shows that entrapped therapeutics would take some time to diffuse out. Summarizing the above results, it can be observed that PβAE-447 showed significant swelling degree with respect to its dry discs.

### Cytotoxic Activity

HEK-293 cell line is commonly used as a first-level screening host to evaluate new transfection vectors. The MTT assay is widely used as a first-level indicator of cytotoxicity. MTT assays help to determine the influence of the added substances on cell proliferation and metabolism. PβAE-447 cytotoxicity in different dosages (10, 20, 30, 40, 50, 60, 75, 90, and 100 μg/ml) was investigated against HEK-293, BEAS-2B, and A549 cells through MTT assay.

**FIGURE 5:**
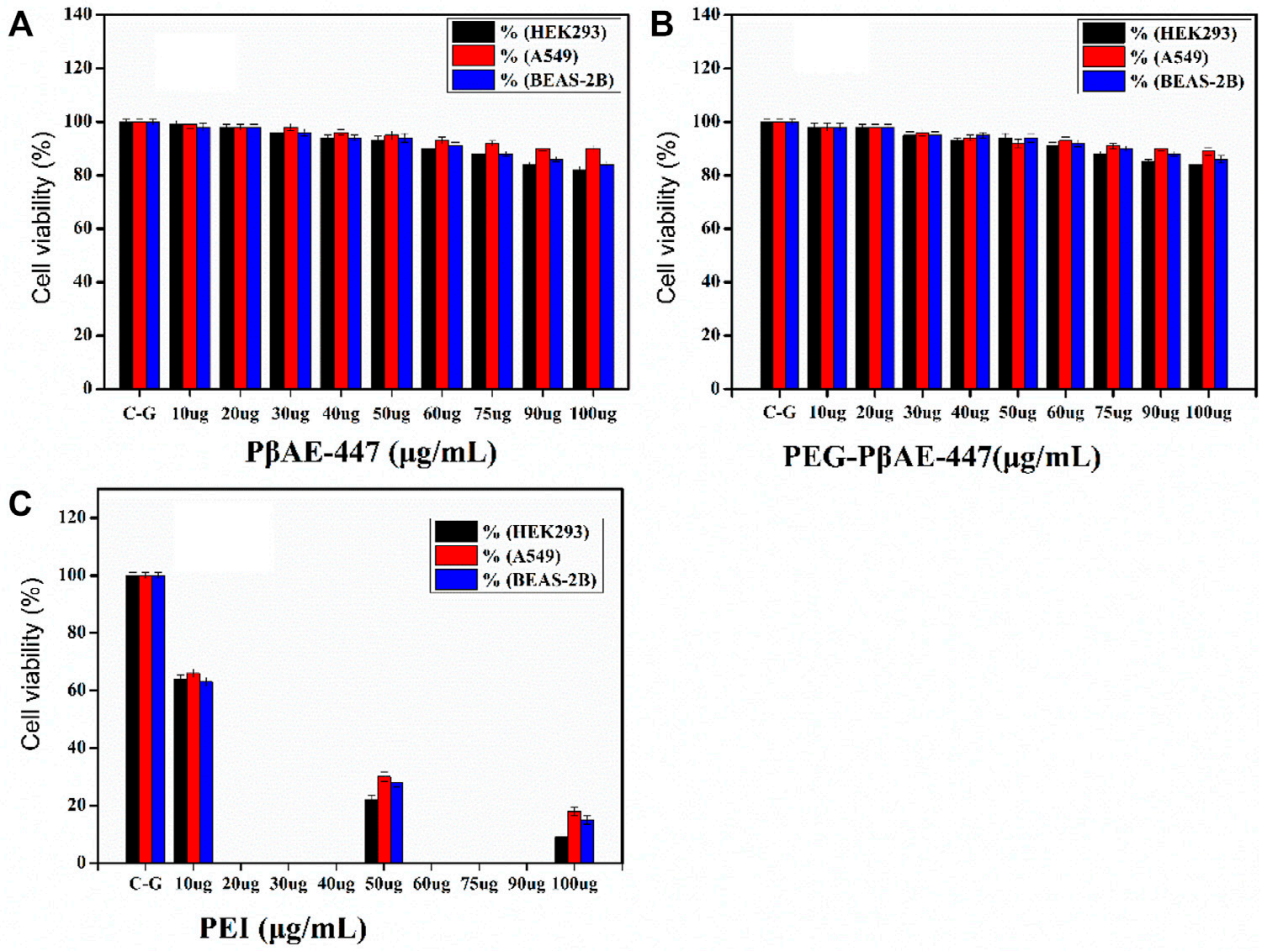
Cell viability assay for pure and PEGYlated PβAE-447 at various concentrations towards HEK-293, BEAS-2B, and A549 cells. PEI (25 kDa) was used as a control (n = 3; and error bars represent standard deviation).

As shown in Figure 5, cells incubated with pure (Figure 5A) and PEGylated PβAE-447 (Figure 5B) show a cell viability sketch. For example, the cell viability of only PβAE-447 is higher than 80% for A549, 77% for BEAS-2B, and 76% for HEK-293 cells. The polymer is cytocompatible toward the investigated cell lines. We did not observe any significant difference in the cytotoxicity results by the conjugation of shielding polymer (PEG) to PβAE-447. PEG has been known as a safe, inert, and non-immunogenic synthetic polymer (Richter et al., 2021). The results are significantly better than the data obtained from the cells population treated with PEI (Mw ≈ 25 k) (Figure 5C), as a positive control as well as to facilitate the comparison. The PEI results revealed a dose-dependent decrease in cells viability. One of the remarkable decreases in cell viability was noted in the PEI treated group at a concentration of 100 µg/ml. Overall, PEI has a higher cytotoxic effect than PβAE-447. The cell viability percentage was (14 ± 5%) for PEI and (86 ± 4%) for PβAE-447 at 100 μg/ml concentration. High molecular weight PEI has a strong positive charge and is more cytotoxic than low molecular weight PEI (Valente et al., 2021).

On the other hand, PβAE-447 showed a dose-dependent decrease in cell viability. However, the cell viability was slightly reduced as the PβAE-447 concentration was raised; despite, the cell viability was higher than 75% for all 3 cell lines, confirming that the PβAE-447 is cytocompatible. Disulfide bonds in the PβAE support biodegradability, influencing vector cytotoxicity and pDNA release (Liu et al., 2019).

### Nanoparticles Characterization

To study the particle size and zeta potential as potential variables, the nanoparticles were characterized as: 1) nanoparticles formed with 10% FBS; 2) nanoparticles formed with 300 mmol NaCl; 3) lyophilized nanoparticles; 4) PEGYlated nanoparticles in FBS, and 5) PEGYlated nanoparticles with 300 mmol NaCl. The obtained results were compared with the fresh nanoparticle properties (the as-prepared material). All these results were evaluated with the nanoparticles prepared at a 60:1 polymer: pDNA weight ratio.

**FIGURE 6:**
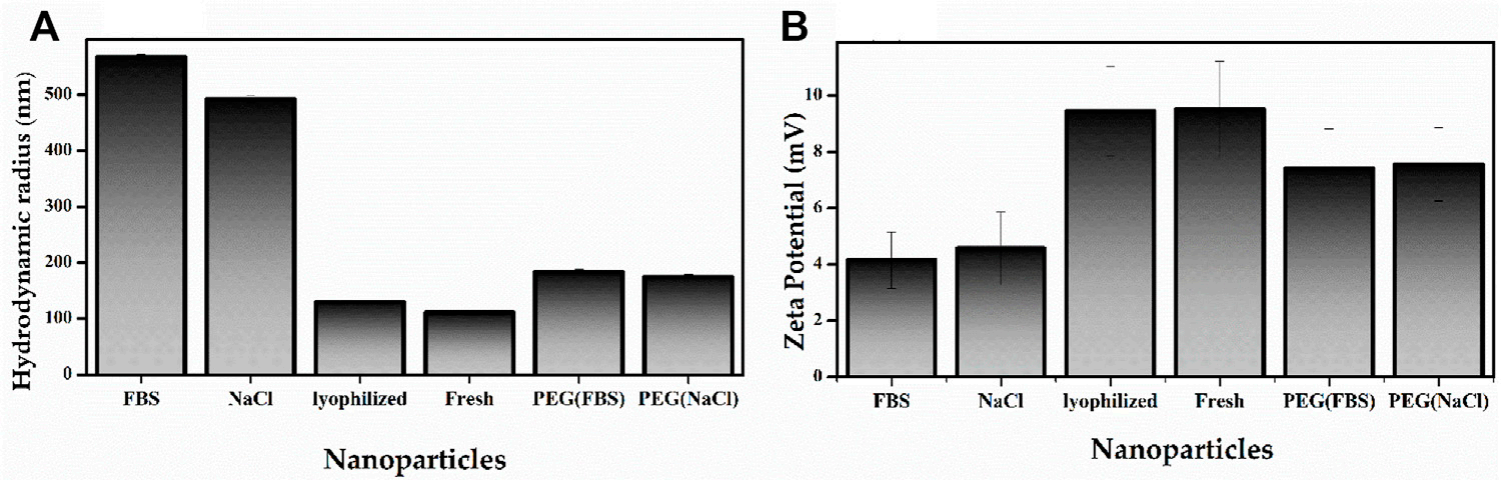
Characterization studies of PβAE-447/pDNA nanoparticles; Nanoparticle Size **(A)** and Zeta potential **(B)** of the as-prepared PβAE-447/pDNA nanoparticles in FBS and NaCl after 4 hours at room temperature, Lypholized, as-prepared and PEGylated PβAE-447/pDNA nanoparticles in FBS and NaCl.

The size of the as-prepared material was 111.1 nm while the lyophilized nanoparticle’s size remained 129.9 nm after 4 months of storage (Figure 6A and Supplementary Figure S3). This size range is suitable for cellular uptake. It has been reported that nanoparticles between 20 and 200 nm can internalize in cells, acting as target nanocarriers. In this size range, the nanoparticles can prevent filtration and quickly penetrate the cells (Zhao et al., 2014).

The Zeta potential of the as-prepared material was 9.51 mV while the lyophilized nanoparticle’s Zeta potential remained 9.45 mV nm after 4 months of storage. The Zeta potential is directly associated with the surface charge. So, it is critical for nanoparticle stability in physiological media and responsible for the initial nanoparticle adsorption on the cell membrane. After adsorption, the endocytic uptake rate is influenced by the particle’s size (Green et al., 2008; Rasmussen et al., 2020). These statements indicate that nanoparticle sizes affect cellular internalization, transfection efficiency, and biodistribution *in vivo*.

The PβAE/pDNA nanoparticles incubated with FBS and NaCl were aggregated, leading to large-sized particles with low Zeta potentials (Figure 6A,B and Supplementary Figure S3).

The FBS accelerates the nanoparticle aggregation, indicating that the PβAE/pDNA nanoparticle zeta potential is quickly altered by the adsorption of serum proteins. The FBS adsorption increased the average nanoparticle size to 567 nm after 4 h (Figure 6A). For preventing or reducing nanoparticle aggregation, the PβAE-447 was PEGylated before nanoparticle formulation. PEGylated polymers have a lower affinity to blood proteins because PEG is not charged (Kim et al., 2015). Compared to PβAE/pDNA nanoparticles in FBS and NaCl, the PEG adsorption reduced the PEGylated-PβAE/pDNA nanoparticles hydrodynamic radius to 184 nm in FBS and 175.1 nm in NaCl after 4 h (Figure 6). The size and Zeta potential of the PEGylated-PβAE-447/p-DNA nanoparticles in FBS and NaCl after 4 h were different compared to the as-prepared PβAE-447/p-DNA nanoparticles, confirming strong evidence of successful PEG coating. The PEGylated polymer reduced and prevented nonspecific interactions between the nanoparticles and FBS, avoided aggregation.

Non-PEGylated PβAE-based nanoparticle suspensions are unstable in NaAc solution, forming aggregates during long-term storage at room temperature (Wilson et al., 2019). For overcoming this disadvantage, the PβAE/pDNA nanoparticles were lyophilized and stored at 4 °C for 4 months. These nanoparticles were re-suspended in distilled water, and their average size and Zeta potential were measured and compared with the properties found for the fresh nanoparticles (as-prepared materials). The lyophilization and storage process at 4 °C for 4 months did not influence the nanoparticle features. The average sizes and Zeta potentials of the lyophilized and fresh nanoparticles are similar (Figure 6). These properties are desirable for gene delivery and cellular uptake. Therefore, the formulations that developed significant aggregations were eliminated for further considerations as candidates for gene delivery. The as-prepared PβAE-447/pDNA nanoparticles, lyophilized, and PEGylated nanoparticles were selected for further studies.

**FIGURE 7:**
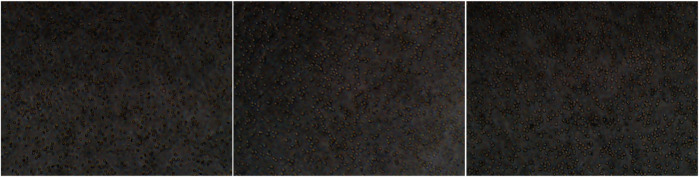
Agglutination with erythrocytes **(**scale = 50 μm n = 3).

Various cationic non-viral gene vectors have shown agglutination activities (Kurosaki et al., 2009). Hemagglutination assay with mice erythrocytes was performed to investigate the agglutinating activity of PEGylated PβAE-447/pDNA complexes at a 60 wt/wt ratio. Subsequently, 2% erythrocytes suspension was selected because it supports best agglutination observation in the 96-well plate (Mrázková et al., 2019). Before using PEGylated PβAE-447/pDNA complexes for clinical applications, it is necessary to investigate its biocompatibility with blood components. The hemagglutination assay results ensure the PEGylated PβAE-447/pDNA complexes are cytocompatible with blood cells, as no RBC disruption was observed (Figure 7).

### Cell Transfection

The transfection efficiency of the nanoparticles was investigated toward three different cell lines (HEK-293, BEAS-2B, and A549). HEK-293 cells are preferred as an easily transfectable cell line, while BEAS-2B and A549 cells are selected as relevant target cell lines for transfection. These cells were seeded in 96-well plates at 12,500 cells/well in 100 μL of media and were incubated overnight for adherence. The pDNA concentration (0.06 μg/μL) in each well was kept constant.

As a model system, the same protocols were followed in each transfection experiment by using pGFP as a reporter gene. The cells were incubated for 4 h with the nanoparticles (as prepared, lyophilized, and PEGlayted) and cellular uptake was investigated after 48 h. The transfection efficiency was calculated from the microscopic images by analyzing the fraction of stained cells (green color).

**FIGURE 8:**
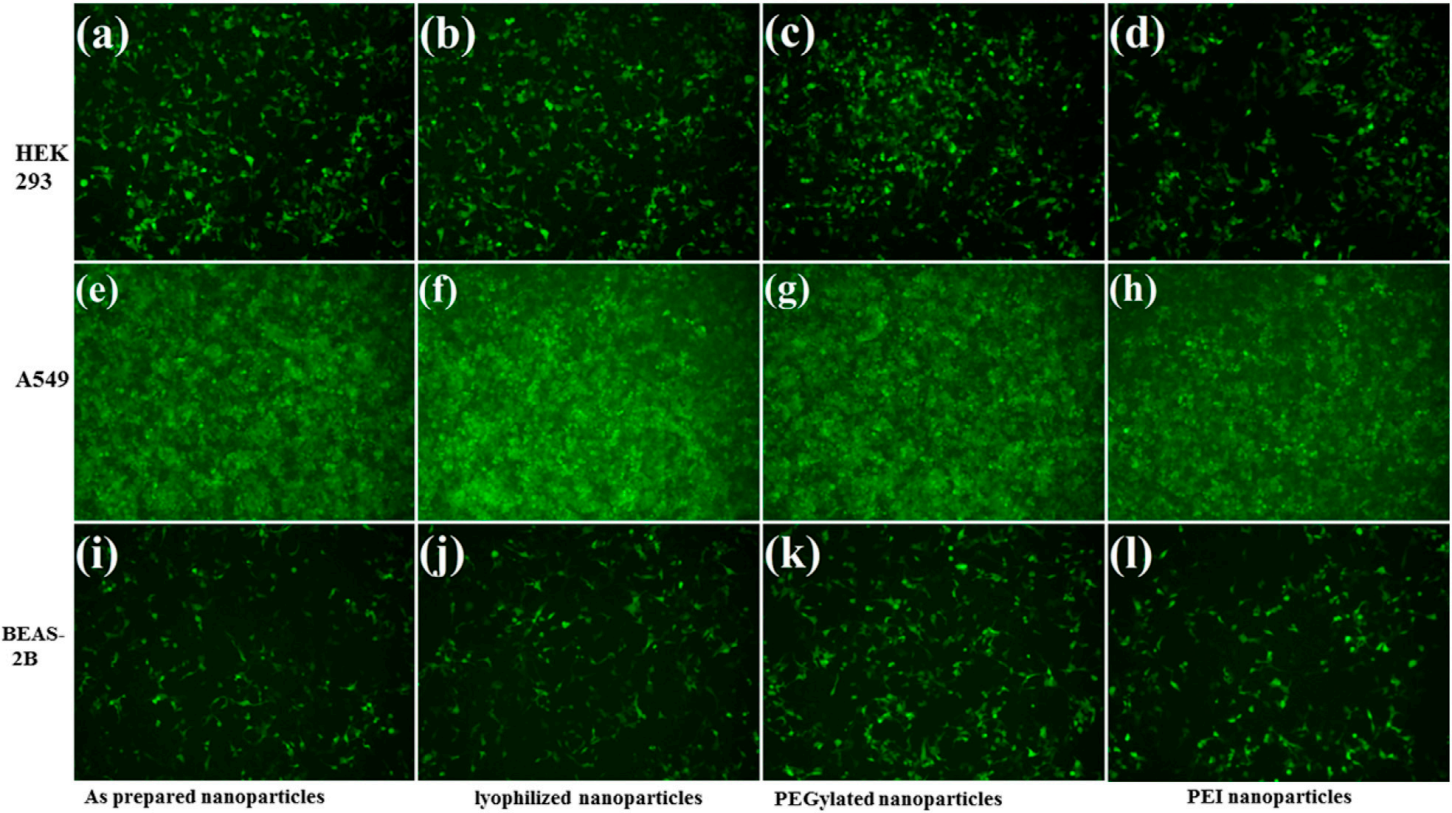
Transfection efficiency of PβAE-447/pDNA nanoparticles in different cells after incubation for 4 hours with as-prepared PβAE-447/pDNA nanoparticles **(A,E,I)**, lyophilized PβAE-447/pDNA nanoparticles **(B,F,J)**, PEGylated PβAE-447/pDNA nanoparticles **(C,G,K)** and PEI **(D,H,L)**.

Fluorescence images demonstrate that transfection efficiency considerably depends on the cell line (Figure 8). The A549 and HEK-293 cells show more GFP expression than the BEAS-2B. The A549 cells have been well transfected by PβAE-447-based nanoparticles, followed by the HEK-293 cells. However, the BEAS-2B cells are less transfected. Overall, PEI was found to have slightly lower transfection efficiency than PβAE-447-based nanoparticles. These results are consistent with the sunshine et al., findings. Their optimal formulation of PβAE showed better transfection in retinal pigment epithelium cells than PEI (25 kDa) (Sunshine et al., 2012). In contrast to PEI, Poly (β-amino ester)s have demonstrated higher transfection efficacy *in vitro* and *in vivo* than many commercially available transfection reagents. PβAEs can deliver various pDNA to multiple tumor models with improved survival outcomes (Wilson et al., 2019). The transfection efficiency is not solely dependent on the PβAE nanoparticles. Additional parameters such as the number of cells, transfection method, and transfection reagents influence the transfection efficiency of a DDS.

**FIGURE 9:**
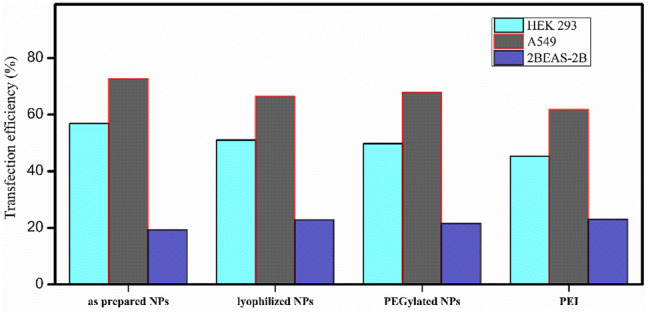
Transfection efficiencies of PβAE-447 and PEI toward A-549, HEK 293, and BEAS-2B were calculated by ImageJ (n = 3).

No significant difference in the transfection efficiency is observed among the lyophilized PβAE-447/pDNA, PEGlated PβAE-447/pDNA, and as-prepared PβAE-447/pDNA nanoparticles (Figure 9). The highest transfection efficiency (72%) was shown by the as-prepared PβAE-447/pDNA nanoparticles toward A549 cells, followed by the HEK-293 cells (56%) at a 60 PβAE-447/pDNA weight ratio. Interestingly, we found that PEI has slightly high transfection in BEAS-2B cells. We assume that different levels of transfection of the same reagents may be due to variant specificity toward target cells.

### Confocal Microscopy

This experiment explores the capability of PβAE-447 to deliver pDNA to the nucleus of the cells. HEK-293, BEAS-2B, and A549 cells were treated only with PEGYlated PβAE-447/pDNA nanoparticles at different times to evaluate the endocytosis and internalization. Endocytosis is the essential cellular process in which the extracellular materials and nanoparticles are internalized in cells (Rennick et al., 2021). The confocal images show that PEGYlated PβAE/pDNA nanoparticles are taken up by the evaluated cell lines.

**FIGURE 10:**
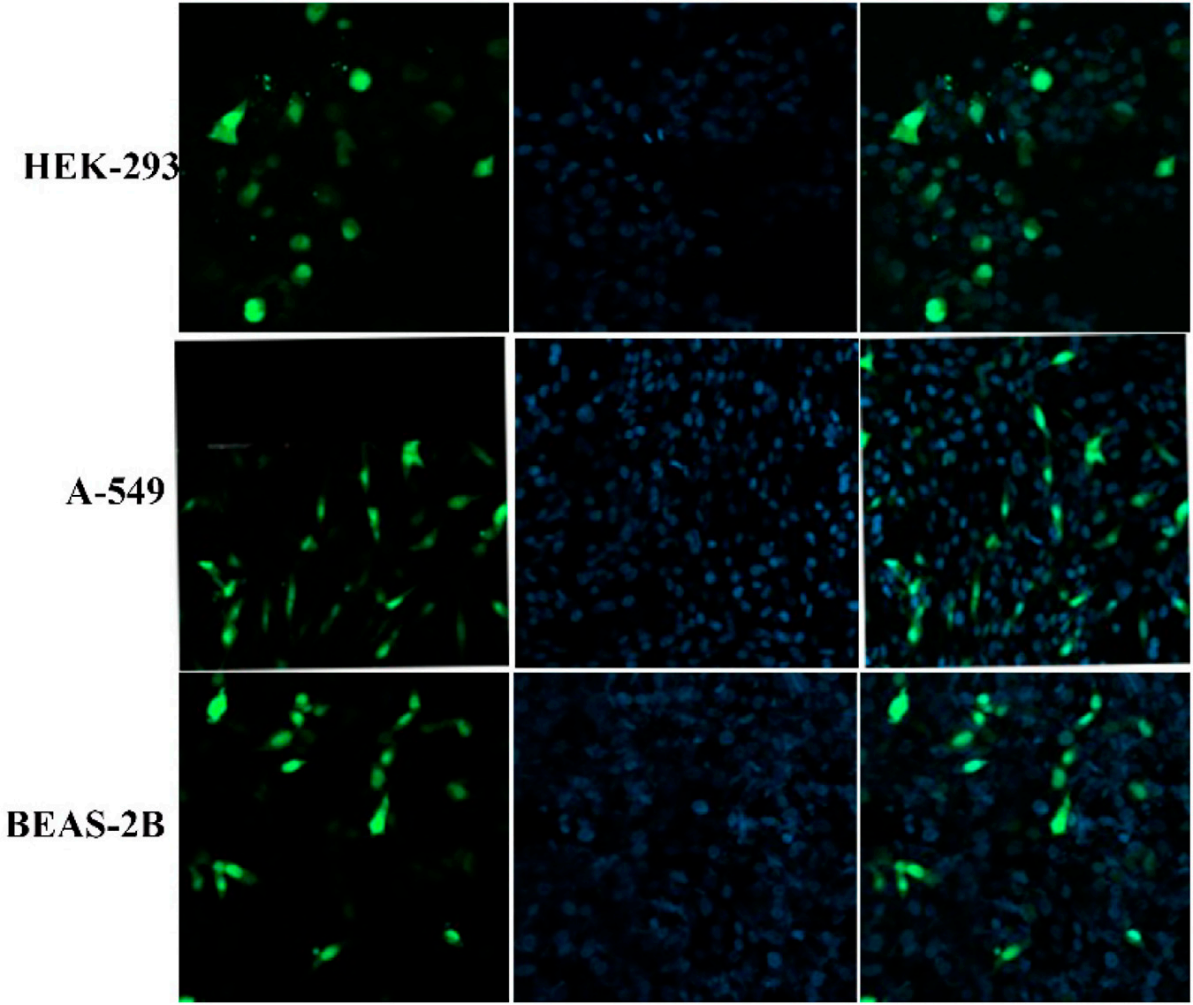
Confocal microscopy images showed the cellular uptake of PEGylated nanoparticles after 4 h of incubation.

The results demonstrate that the short incubation period (at least 2 h) did not show significant cellular uptake, while long incubation periods (6 and 8 h) led to high cytotoxic effects. This indicates that polymer has a harmful effect on cells if exposed for a longer time. This toxic effect may depend on the charge of the PβAE-447. Meantime, when PβAE-based nanoparticles were incubated with the cells for 4 h, the nanoparticles are found primarily distributed in the cytoplasm and nuclei region (Figure 10). It indicates that PβAE-based nanocomplexes can be used to target the nuclei effectively. However, long exposure times provide cytotoxic effects, because the strong influence of high charges may lyse the membranes. Long time disrupting the membrane cell walls fluidity due to the increased cell permeability and degeneration (Jeong et al., 2017).

## Discussion

Nanoparticles for therapeutic delivery have been a subject of research. However, many obstacles have to be addressed before clinical practice. The peculiar mechanism by which polymeric nanoparticles mediate specificity remains unclear. Primarily, it relies on the nature and structure of the polymer used to design the nanoparticles (Patra et al., 2018). The nanoparticle’s properties depend on the polymer features, including the molecular mass, solubility, cytocompatibility, biodegradability, pH-responsiveness, etc. For promoting enhanced gene transfection, a biodegradable and linear PβAE-447 was synthesized and characterized. This polymer was used to design new DDSs with enhanced transfection efficiency toward different cell lines.

Poly (β-amino ester)s have been demonstrated as safe and efficient transfection vectors *in vitro* for various cell types. PβAEs can easily be formulated to selectively target desired tissues while avoiding nearby healthy tissues (Mangraviti et al., 2015). Various strategies can be applied for modulating the polymer properties to design DDSs for efficient transfection. The monomer concentration ratios result in significant changes in the polymerization kinetic rates (Safaei et al., 2021). The PβAE-447 synthesis proceeded through a conjugate addition reaction. The polymerization resulted in a broad molecular weight, indicating polymer lengths with chain end groups and molecular variant polymer dependent on the monomers’ stoichiometric ratio during synthesis. An excess quantity of either monomer (diacrylate or amine) predominates acrylate or amine-terminated polymers, respectively. Theoretically, high molecular weight PβAEs are yielded in the stoichiometric equivalence of the monomers (Anderson et al., 2005). Therefore, monomers’ molar ratio of 1:1 is essential to promote a high degree of monomers conversion to prepare homogeneous polymers with high molecular weights.

PβAE-447 was selected for characterization studies because it is more conducive to efficient gene transfection than other PβAEs. More transfection occurs with B4S4 base polymer with end groups E6 and E7 (Sunshine et al., 2012; Sunshine et al., 2012; Tzeng and Green 2013). Polymers fabricated with E9 as an end group demonstrated significant cytotoxicity, while polymers formulated with E10 and E12 showed poor transfection performance (Sunshine et al., 2011). Besides varying monomers, the synthesis conditions and monomer ratios can also be altered to develop a vast library of polymers with diverse structures and applications (Rui et al., 2017). In addition, many different types of amines and diacrylates (Supplementary Figure S1) can be used at wide monomer ratios to synthesize PβAEs with various properties. However, the most significant factor for efficient transfection is the effect of end-capped groups on polymer performance (Iqbal et al., 2020).

The aqueous carrier solubility is an important requirement in the DDSs selection process. The solubility of polymer helps to determine the hydrophobic/hydrophilic nature of projected DDSs. The dissociation of the genetic materials from nanoparticles can be controlled by modulating the solubility of the carrier polymer concerning the external environment. Many publications recommended mixing equal volumes buffer system containing pDNA and transfection reagents, followed by an incubation period for complexation formation (Engelhardt et al., 2017). We hypothesize that partial PβAE-447 solubility in NaAc might influence the nanocomplex formation with pDNA before adding to the cells. Therefore, knowledge of polymer dissolution behavior is critical in understanding time-released applications.

The polymer buffering capacity is considered an essential parameter for polymeric vectors, regulating the pDNA release from the polymer matrix inside the cells after endocytosis. Amine moieties on the polymers can be protonated and deprotonated depending on the pH. Protonated amines increase the osmotic pressure inside endosomes, disrupting the endosomal membrane, leading the polymer matrix to escape (Bhise et al., 2010). The high buffering capacity of carriers contributes to the gene transfection, protecting the pDNA structure after endocytosis (Kim et al., 2013). Protonated polymers stabilize the negatively pDNA by electrostatic interactions. Therefore, understanding the proton buffering capacity of the PβAEs is essential not only because it is evolving non-viral vectors but also, because this knowledge may help to synthesize and design next-generation non-viral vectors.

The swelling property influences the drug diffusion and releases behavior from DDSs (Wang et al., 2010). Hydrophilic DDSs swell because of water diffusion and uptake. The adsorbed solvents interact with the drug, supporting the release of solid solutes from DDSs (Sienkiewicz et al., 2017). Cationic polymers have amino groups that can be protonated depending on the pH condition and pK_a_ of the protonated sites. Protonated amines interact better with solvent molecules than unprotonated amines. Ion-dipole interactions between solvent and cationic polymers increase swelling due to increased solvent solvation capacity toward the charged polymer structure (Deen and Loh 2018).

The PβAE-447 vector is cytocompatible, distinguishing it from other cationic vectors in terms of degradability in the physiological environment (Green et al., 2009). In contrast to non-degradable PEI-based polymers, PβAEs contain tertiary amines that facilitate rapid aqueous hydrolysis of the polymer backbone. This feature designates PβAE with generally low cytotoxicity and effectively no risk of accumulation following repeat administration *in vivo* (Wilson et al., 2019)*.* All these findings ensure that PβAE-447 is biodegradable and that the degraded byproducts are also highly cytocompatible without causing any prominent cytotoxicity during the gene transfection experiment. Nonetheless, the excellent cell viability profile of PβAE-447 suggests that they are interested candidates for further study as pDNA condensing agents.

The size and zeta potential of DDS are the most critical parameters that regulate the therapeutic effects of nanoparticles. These features strongly affect the systemic circulation and stability of the DDS in the body and its cellular uptake by the cells. The binding affinity between pH-responsive PβAEs and p-DNA is critical for nanoparticle formation and p-DNA release due to pH alteration (Bishop et al., 2013). DDSs containing protonated amino groups interact better with negatively charged cells, supporting the DDS internalization. However, it is challenging to optimize the size and zeta potential of DDSs to improve carriers’ efficacy.

Determination of cellular uptake or internalization is the most critical physicochemical parameter that must be evaluated before *in vivo* applications. The nanoparticle internalization should occur through endocytosis in many cells; however, only sub-micron-sized nanoparticles are effectively uptaken in the HepG2, Hepa one to six, and KLN205 cells (Nimesh 2012). The nanoparticle characterization under physiological conditions is critical and challenging. The surrounding environment such as the medium (solvent, body fluids, etc.), ionic strength, charged macromolecules (e.g., proteins) strongly influences the nanoparticle properties for example size, and charge density. The physiological fluids are composed of proteins that interact with charged nanoparticles, forming a “protein corona.” This behavior affects the nanoparticle properties, altering its size, shape, and charge density (Van Hong Nguyen 2017). Besides, the polymer/pDNA nanocomplexes also naturally tend to aggregate in physiological fluids (Xia et al., 2011). In addition, the nanoparticles’ aggregate as the Zeta potential is lower than +30 mV and higher than −30 mV because the attractive forces may exceed the repulsive forces provided by charged materials. Nanoparticles with Zeta potential higher than +30 mV can resist more against aggregation in solution, being electrically stable (Hwangbo et al., 2021). Therefore, the polyplexes’ stability in the physiological environment containing FBS and NaCl was investigated by evaluating the size and Zeta potential of the nanoparticles over 4 h of incubation.

The lower Zeta potential is thought to be because of 10% FBS in the culture medium, which facilitates the attraction of negatively charged albumin that interacts with positively charged nanoparticles at neutral pH. Ions can adsorb on the nanoparticle surface, modify the magnitude of zeta potential. The decrease of zeta potential facilitates a closer approach of nanoparticles, and boost aggregations (Shrestha et al., 2020). In sum, the results show that the presence of FBS and NaCl are the leading factors to aggregate the PβAE/p-DNA nanoparticles, modifying their sizes. Besides, the non-specific interactions in physiological media may cause particle aggregation and opsonization, thereby reducing the systemic circulation time.

The lyophilized nanoparticles preserved their properties and efficacy after 4 months’ storage. This result is remarkably important as it assures the stability, scalability, and robustness of the lyophilized particles over time. Corresponding to maintaining colloidal stability, the nanoparticles formulated with PEG-PβAE-447 displayed significantly better *in vitro* transfection efficiency. These findings revealed that PEGylated NPs retained *in vitro* transfection efficiency and stability compared to non-PEGylated NPs. These findings support that the PβAE-447/pDNA nanoparticles can be used as efficient gene delivery vectors, being exciting devices for further studies *in vivo*.

Successful transfection efficiency is also influenced by the cell type. Since different cells are likely to behave differently to the same transfection reagent, hence choosing an appropriate cell type is necessary to maximize results (Neuhaus et al., 2016). In summary, the positively charged PβAE/pDNA nanoparticles are effectively attached to the cell membrane (negatively charged) by electrostatic interactions, entering the cells by endocytosis. The protonated amines in the PβAE-447 raise the osmotic pressure inside the endosome, disrupting the endosomal membrane. Thus, the PβAE/pDNA nanoparticle internalizes, leading to gene expression in host cells (Iqbal et al., 2020).

Besides the incubation period, other factors such as nanoparticle properties (size, shape, zeta potential), cellular microenvironment, and experimental factors (temperature) significantly affect the intracellular fate of nanoparticles. Behzadi et al. mentioned that small-sized nanoparticles internalized in cells faster than large particles (Behzadi et al., 2017). Some nanocarriers cannot reach the cell nuclei of transfected cells, suppressing the transfection. PβAE-447 is a capable carrier to enter the cell, penetrate the nucleus and release some portion of the complexed pDNA, thus making it available for cell transfection.

To achieve the best possible formulation to display the highest stability, biocompatibility, and transfection efficiency, a series of PβAE/pDNA nanoparticles were synthesized and evaluated. PEG- PβAE/pDNA nanoparticles were found to be the best formulation which confirmed the optimal balance of all the parameters i.e. (size, zeta potential and colloidal stability). Hence, it exhibited the highest transfection efficacy with low toxicity. The outcomes of this work could be further used in multidisciplinary fields of cationic polymers, to design and fabricate a new generation of nanoparticle-based delivery systems for gene therapy and gene editing applications.

## Conclusion

Characterizations studies open a gateway in improving vectors’ design and architecture to increase cargo-carrying capacity, advance target specificity, and improve biodegradability, the basic requirements for successful gene therapy. In summary, PβAE-447 was successfully synthesized and various parameters were characterized to improve its robustness. The exhibition of the high buffering capacity of PβAE-447 at acidic pH would help early endosomal escape. Lyophilized nanoparticles maintained appropriate size, Zeta potential, and transfection activity after four 4 months of storage. First, it was revealed that PβAE-447/pDNA nanocomplexes tended to form aggregates in presence of serum and ions. As a result of PEG conjugation, the colloidal stability of nanocomplexes was improved. The transfection efficiency of PEGylated and lyophilized nanoparticles are better than PEI, particularly in A549 cells. These results reveal for the first time the importance of optimizations in the formulation process of PβAE-447. The results presented here can facilitate further investigation to fabricate and optimize DDSs for higher performance *in vivo.*

## Data Availability

The original contributions presented in the study are included in the article/Supplementary Material, further inquiries can be directed to the corresponding author.

## References

[B1] Agarwal S. Zhang Y. Maji S. Greiner A. (2012). PDMAEMA Based Gene Delivery Materials. Mater. Today 15, 388–393. 10.1016/s1369-7021(12)70165-7

[B2] Anderson D. G. Akinc A. Hossain N. Langer R. (2005). Structure/property Studies of Polymeric Gene Delivery Using a Library of Poly(beta-Amino Esters). Mol. Ther. 11, 426–434. 10.1016/j.ymthe.2004.11.015 15727939

[B3] Behzadi S. Serpooshan V. Tao W. Hamaly M. A. Alkawareek M. Y. Dreaden E. C. (2017). Cellular Uptake of Nanoparticles: Journey inside the Cell. Chem. Soc. Rev. 46, 4218–4244. 10.1039/c6cs00636a 28585944 PMC5593313

[B4] Bhise N. S. Gray R. S. Sunshine J. C. Htet S. Ewald A. J. Green J. J. (2010). The Relationship between Terminal Functionalization and Molecular Weight of a Gene Delivery Polymer and Transfection Efficacy in Mammary Epithelial 2-D Cultures and 3-D Organotypic Cultures. Biomaterials 31, 8088–8096. 10.1016/j.biomaterials.2010.07.023 20674001 PMC3175420

[B5] Bishop C. J. Ketola T. M. Tzeng S. Y. Sunshine J. C. Urtti A. Lemmetyinen H. (2013). The Effect and Role of Carbon Atoms in Poly(β-Amino Ester)s for DNA Binding and Gene Delivery. J. Am. Chem. Soc. 135, 6951–6957. 10.1021/ja4002376 23570657 PMC3838887

[B6] Biswal D. Wattamwar P. P. Dziubla T. D. Hilt J. Z. (2011). A Single-step Polymerization Method for Poly(β-Amino Ester) Biodegradable Hydrogels. Polymer 52, 5985–5992. 10.1016/j.polymer.2011.10.058

[B7] Chen C. K. Huang P. K. Law W. C. Chu C. H. Chen N. T. Lo L. W. (2020). Biodegradable Polymers for Gene-Delivery Applications. Int. J. Nanomedicine 15, 2131–2150. 10.2147/IJN.S222419 32280211 PMC7125329

[B8] Deen G. R. Loh X. J. (2018). Stimuli-responsive Cationic Hydrogels in Drug Delivery Applications. Gels 4, 13. 10.3390/gels4010013 PMC6318685 30674789

[B9] Engelhardt K. H. Pinnapireddy S. R. Baghdan E. Jedelská J. Bakowsky U. (2017). Transfection Studies with Colloidal Systems Containing Highly Purified Bipolar Tetraether Lipids from Sulfolobus Acidocaldarius. Archaea.2017. 10.1155/2017/8047149 PMC5292391 28239294

[B10] Ferrell W. H. Kushner D. I. Hickner M. A. (2017). Investigation of Polymer-Solvent Interactions in Poly(styrene Sulfonate) Thin Films. J. Polym. Sci. Part. B: Polym. Phys. 55, 1365–1372. 10.1002/polb.24383

[B11] Gong J. H. Wang Y. Xing L. Cui P. F. Qiao J. B. He Y. J. (2018). Biocompatible Fluorinated Poly(β-Amino Ester)s for Safe and Efficient Gene Therapy. Int. J. Pharm. 535, 180–193. 10.1016/j.ijpharm.2017.11.015 29129572

[B12] Gonçalves G. A. R. Paiva R. M. A. (2017). Gene Therapy: Advances, Challenges and Perspectives. Einstein (Sao Paulo). 15, 369–375. 29091160 10.1590/S1679-45082017RB4024 PMC5823056

[B13] Green J. J. Langer R. Anderson D. G. (2008). A Combinatorial Polymer Library Approach Yields Insight into Nonviral Gene Delivery. Acc. Chem. Res. 41, 749–759. 10.1021/ar7002336 18507402 PMC3490629

[B14] Green J. J. Zugates G. T. Langer R. Anderson D. G. (2009). “Poly(β-amino Esters): Procedures for Synthesis and Gene Delivery,” in Macromolecular Drug Delivery (Springer), 53–63. 10.1007/978-1-59745-429-2_4 PMC4059050 19085119

[B15] Helaly F. M. Hashem M. S. (2013). Preparation and Characterization of Poly (β-Amino Ester) Capsules for Slow Release of Bioactive Material. J. Encapsulation Adsorption Sci. 2013. 10.4236/jeas.2013.33008

[B16] Hwang H. S. Hu J. Na K. Bae Y. H. (2014). Role of Polymeric Endosomolytic Agents in Gene Transfection: a Comparative Study of poly(L-Lysine) Grafted with Monomeric L-Histidine Analogue and poly(L-Histidine). Biomacromolecules 15, 3577–3586. 10.1021/bm500843r 25144273 PMC4195522

[B17] Hwangbo S. A. Kwak M. Kim J. Lee T. G. (2021). Novel Surfactant-free Water Dispersion Technique of TiO2 NPs Using Focused Ultrasound System. Nanomaterials (Basel) 11, 427. 10.3390/nano11020427 33567644 PMC7915381

[B18] Iqbal S. Qu Y. Dong Z. Zhao J. Khan A. R. Rehman S. (2020). Poly (β‐Amino Esters) Based Potential Drug Delivery and Targeting Polymer; an Overview and Perspectives. Eur. Polym. J. 110097.

[B19] Iqbal S. Zhao Z. (2022). Poly (β Amino Esters) Copolymers: Novel Potential Vectors for Delivery of Genes and Related Therapeutics. Int. J. Pharm. 611, 121289. 10.1016/j.ijpharm.2021.121289 34775041

[B20] Jeong H. Hwang J. Lee H. Hammond P. T. Choi J. Hong J. (2017). *In Vitro* blood Cell Viability Profiling of Polymers Used in Molecular Assembly. Sci. Rep. 7, 9481–9513. 10.1038/s41598-017-10169-5 28842713 PMC5573391

[B21] Kamat C. D. Shmueli R. B. Connis N. Rudin C. M. Green J. J. Hann C. L. (2013). Poly(β-amino Ester) Nanoparticle Delivery of TP53 Has Activity against Small Cell Lung Cancer *In Vitro* and *In Vivo* . Mol. Cancer Ther. 12, 405–415. 10.1158/1535-7163.MCT-12-0956 23364678 PMC3624031

[B22] Kim J. Sunshine J. C. Green J. J. (2014). Differential Polymer Structure Tunes Mechanism of Cellular Uptake and Transfection Routes of Poly(β-Amino Ester) Polyplexes in Human Breast Cancer Cells. Bioconjug. Chem. 25, 43–51. 10.1021/bc4002322 24320687 PMC4016154

[B23] Kim J. Wilson D. R. Zamboni C. G. Green J. J. (2015). Targeted Polymeric Nanoparticles for Cancer Gene Therapy. J. Drug Target. 23, 627–641. 10.3109/1061186X.2015.1048519 26061296 PMC4696040

[B24] Kim J. Mondal S. K. Tzeng S. Y. Rui Y. Al-Kharboosh R. Kozielski K. K. (2020). Poly(ethylene Glycol)-Poly(beta-Amino Ester)-Based Nanoparticles for Suicide Gene Therapy Enhance Brain Penetration and Extend Survival in a Preclinical Human Glioblastoma Orthotopic Xenograft Model. ACS Biomater. Sci. Eng. 6, 2943–2955. 10.1021/acsbiomaterials.0c00116 33463272 PMC8035708

[B25] Kim T.-H. Choi H. Yu G. S. Lee J. Choi J. S. (2013). Novel Hyperbranched Polyethyleneimine Conjugate as an Efficient Non-viral Gene Delivery Vector. Macromol. Res. 21, 1097–1104. 10.1007/s13233-013-1154-y

[B26] Kurosaki T. Kitahara T. Kawakami S. Nishida K. Nakamura J. Teshima M. (2009). The Development of a Gene Vector Electrostatically Assembled with a Polysaccharide Capsule. Biomaterials 30, 4427–4434. 10.1016/j.biomaterials.2009.04.041 19473696

[B27] Li C. Tzeng S. Y. Tellier L. E. Green J. J. (2013). (3-aminopropyl)-4-methylpiperazine End-Capped Poly(1,4-Butanediol Diacrylate-Co-4-Amino-1-Butanol)-Based Multilayer Films for Gene Delivery. ACS Appl. Mater. Inter. 5, 5947–5953. 10.1021/am402115v PMC3838882 23755861

[B28] Liu S. Gao Y. Zhou D. Zeng M. Alshehri F. Newland B. (2019). Highly Branched poly(β-Amino Ester) delivery of Minicircle DNA for Transfection of Neurodegenerative disease Related Cells. Nat. Commun. 10, 3307–3314. 10.1038/s41467-019-11190-0 31341171 PMC6656726

[B29] Mangraviti A. Tzeng S. Y. Kozielski K. L. Wang Y. Jin Y. Gullotti D. (2015). Polymeric Nanoparticles for Nonviral Gene Therapy Extend Brain Tumor Survival *In Vivo* . ACS nano 9, 1236–1249. 10.1021/nn504905q 25643235 PMC4342728

[B30] Mrázková J. Malinovská L. Wimmerová M. (2019). Microscopy Examination of Red Blood and Yeast Cell Agglutination Induced by Bacterial Lectins. PloS one 14, e0220318. 31344098 10.1371/journal.pone.0220318 PMC6657890

[B31] Neuhaus B. Tosun B. Rotan O. Frede A. Westendorf A. M. Epple M. (2016). Nanoparticles as Transfection Agents: a Comprehensive Study with Ten Different Cell Lines. RSC Adv. 6, 18102–18112. 10.1039/c5ra25333k

[B32] Nguyen V. H. Lee B. J. (2017). Protein corona: a New Approach for Nanomedicine Design. Int. J. Nanomedicine 12, 3137–3151. 10.2147/IJN.S129300 28458536 PMC5402904

[B33] Nimesh S. (2012). Potential Implications of Nanoparticle Characterization on *In Vitro* and *In Vivo* Gene Delivery. Ther. Deliv. 3, 1347–1356. 10.4155/tde.12.110 23259252

[B34] Patra J. K. Das G. Fraceto L. F. Campos E. V. R. Rodriguez-Torres M. D. P. Acosta-Torres L. S. (2018). Nano Based Drug Delivery Systems: Recent Developments and Future Prospects. J. Nanobiotechnology 16, 71–33. 10.1186/s12951-018-0392-8 30231877 PMC6145203

[B35] Perni S. Prokopovich P. (2020). Optimisation and Feature Selection of Poly-Beta-Amino-Ester as a Drug Delivery System for Cartilage. J. Mater. Chem. B 8, 5096–5108. 10.1039/c9tb02778e 32412019 PMC7412864

[B36] Rasmussen M. K. Pedersen J. N. Marie R. (2020). Size and Surface Charge Characterization of Nanoparticles with a Salt Gradient. Nat. Commun. 11, 2337–2338. 10.1038/s41467-020-15889-3 32393750 PMC7214416

[B37] Rennick J. J. Johnston A. P. R. Parton R. G. (2021). Key Principles and Methods for Studying the Endocytosis of Biological and Nanoparticle Therapeutics. Nat. Nanotechnol 16, 266–276. 10.1038/s41565-021-00858-8 33712737

[B38] Richter F. Leer K. Martin L. Mapfumo P. Solomun J. I. Kuchenbrod M. T. (2021). The Impact of Anionic Polymers on Gene Delivery: How Composition and Assembly Help Evading the Toxicity-Efficiency Dilemma. J. nanobiotechnology 19, 1–15. 10.1186/s12951-021-00994-2 34579715 PMC8477462

[B39] Rui Y. Quiñones G. Green J. J. (2017). Biodegradable and Bioreducible Poly(beta-Amino Ester) Nanoparticles for Intracellular Delivery to Treat Brain Cancer. Aiche J. 63, 1470–1482. 10.1002/aic.15698

[B40] Safaei A. Terryn S. Vanderborght B. Van Assche G. Brancart J. (2021). The Influence of the Furan and Maleimide Stoichiometry on the Thermoreversible Diels-Alder Network Polymerization. Polymers (Basel) 13, 2522. 10.3390/polym13152522 34372124 PMC8347837

[B41] Shmueli R. B. Sunshine J. C. Xu Z. Duh E. J. Green J. J. (2012). Gene Delivery Nanoparticles Specific for Human Microvasculature and Macrovasculature. Nanomedicine 8, 1200–1207. 10.1016/j.nano.2012.01.006 22306159 PMC3350835

[B42] Shrestha S. Wang B. Dutta P. (2020). Nanoparticle Processing: Understanding and Controlling Aggregation. Adv. Colloid Interf. Sci 279, 102162. 10.1016/j.cis.2020.102162 32334131

[B43] Sienkiewicz A. Krasucka P. Charmas B. Stefaniak W. Goworek J. (2017). Swelling Effects in Cross-Linked Polymers by Thermogravimetry. J. Therm. Anal. Calorim. 130, 85–93. 10.1007/s10973-017-6131-9

[B44] Smith T. T. Stephan S. B. Moffett H. F. McKnight L. E. Ji W. Reiman D. (2017). *In Situ* programming of Leukaemia-specific T Cells Using Synthetic DNA Nanocarriers. Nat. Nanotechnol 12, 813–820. 10.1038/nnano.2017.57 28416815 PMC5646367

[B45] Sunshine J. C. Akanda M. I. Li D. Kozielski K. L. Green J. J. (2011). Effects of Base Polymer Hydrophobicity and End-Group Modification on Polymeric Gene Delivery. Biomacromolecules 12, 3592–3600. 10.1021/bm200807s 21888340 PMC3959121

[B46] Sunshine J. C. Peng D. Y. Green J. J. (2012). Uptake and Transfection with Polymeric Nanoparticles Are Dependent on Polymer End-Group Structure, but Largely Independent of Nanoparticle Physical and Chemical Properties. Mol. Pharm. 9, 3375–3383. 10.1021/mp3004176 22970908 PMC3779641

[B47] Sunshine J. C. Sunshine S. B. Bhutto I. Handa J. T. Green J. J. (2012). Poly(β-amino Ester)-Nanoparticle Mediated Transfection of Retinal Pigment Epithelial Cells *In Vitro* and *In Vivo* . PloS one 7, e37543. 10.1371/journal.pone.0037543 22629417 PMC3357345

[B48] Tzeng S. Y. Green J. J. (2013). Subtle Changes to Polymer Structure and Degradation Mechanism Enable Highly Effective Nanoparticles for siRNA and DNA Delivery to Human Brain Cancer. Adv. Healthc. Mater. 2, 468–480. 10.1002/adhm.201200257 23184674 PMC3838886

[B49] Valente J. F. A. Pereira P. Sousa A. Queiroz J. A. Sousa F. (2021). Effect of Plasmid DNA Size on Chitosan or Polyethyleneimine Polyplexes Formulation. Polymers (Basel) 13, 793. 10.3390/polym13050793 33807586 PMC7962013

[B50] Wang Q. Xie X. Zhang X. Zhang J. Wang A. (2010). Preparation and Swelling Properties of pH-Sensitive Composite Hydrogel Beads Based on Chitosan-G-Poly (Acrylic Acid)/vermiculite and Sodium Alginate for Diclofenac Controlled Release. Int. J. Biol. Macromol 46, 356–362. 10.1016/j.ijbiomac.2010.01.009 20096301

[B51] Wang Y. Wang C.-F. Lie M. Zhou D.-Z. Huang W. Wang W.-X. (2020). Effects of Branching Strategy on the Gene Transfection of Highly Branched Poly(β-Amino Ester)s. Chin. J. Polym. Sci. 38, 830–839. 10.1007/s10118-020-2393-y

[B52] Wilson D. R. Sen R. Sunshine J. C. Pardoll D. M. Green J. J. Kim Y. J. (2018). Biodegradable STING Agonist Nanoparticles for Enhanced Cancer Immunotherapy. Nanomedicine 14, 237–246. 10.1016/j.nano.2017.10.013 29127039 PMC6035751

[B53] Wilson D. R. Suprenant M. P. Michel J. H. Wang E. B. Tzeng S. Y. Green J. J. (2019). The Role of Assembly Parameters on Polyplex Poly(beta-Amino Ester) Nanoparticle Transfections. Biotechnol. Bioeng. 116, 1220–1230. 10.1002/bit.26921 30636286 PMC6690498

[B54] Xia J. Tian H. Chen L. Lin L. Guo Z. Chen J. (2011). Oligoethylenimines Grafted to PEGylated Poly(β-Amino Ester)s for Gene Delivery. Biomacromolecules 12, 1024–1031. 10.1021/bm101361g 21355539

[B55] Yu C. Li L. Hu P. Yang Y. Wei W. Deng X. (2021). Recent Advances in Stimulus‐Responsive Nanocarriers for Gene Therapy. Adv. Sci., 2100540. 10.1002/advs.202100540 PMC8292848 34306980

[B56] Zeb A. Rana I. Choi H. I. Lee C. H. Baek S. W. Lim C. W. (2020). Potential and Applications of Nanocarriers for Efficient Delivery of Biopharmaceuticals. Pharmaceutics 12, 1184. 10.3390/pharmaceutics12121184 PMC7762162 33291312

[B57] Zeng M. Zhou D. Ng S. Ahern J. O. K. Alshehri F. Gao Y. (2017). Highly Branched Poly(5-Amino-1-Pentanol-Co-1,4-Butanediol Diacrylate) for High Performance Gene Transfection. Polymers (Basel) 9, 161. 10.3390/polym9050161 PMC6432012 30970840

[B58] Zhang X. F. Liu Z. G. Shen W. Gurunathan S. (2016). Silver Nanoparticles: Synthesis, Characterization, Properties, Applications, and Therapeutic Approaches. Int. J. Mol. Sci. 17, 1534. 10.3390/ijms17091534 PMC5037809 27649147

[B59] Zhao X. Cui H. Chen W. Wang Y. Cui B. Sun C. (2014). Morphology, Structure and Function Characterization of PEI Modified Magnetic Nanoparticles Gene Delivery System. PLoS One 9, e98919. 10.1371/journal.pone.0098919 24911360 PMC4049641

[B60] Zou W. Liu C. Chen Z. Zhang N. (2009). Preparation and Characterization of Cationic PLA-PEG Nanoparticles for Delivery of Plasmid DNA. Nanoscale Res. Lett. 4, 982–992. 10.1007/s11671-009-9345-3 20596550 PMC2893611

