# Supplementary material for: Synthesis and Characterization of Poly (β-amino Ester) and Applied PEGylated and Non-PEGylated Poly (β-amino ester)/Plasmid DNA Nanoparticles for Efficient Gene Delivery
Source: Front Pharmacol. 2022 Apr 8;13:854859. doi: 10.3389/fphar.2022.854859 (PMC9023864; doi:10.3389/fphar.2022.854859)
Supplement: Supplementary file 1 [file DataSheet1.docx]

Synthesis and Characterization of Poly (β-amino ester) and Applied PEGylated and non-PEGylated Poly (β-amino ester)/plasmid DNA Nanoparticles for Efficient Gene Delivery

Sajid Iqbal^1^, Alessandro F. Martins^2, 3, 4,^ Muhammad Sohail^5^, Jingjing Zhao^1^, Qi Deng^1^, Muhan Li^1^, and Zhongxi Zhao ^1,6,7,8^*

^1^ Department of Pharmaceutics, Key Laboratory of Chemical Biology of Ministry of Education, School of Pharmaceutical Sciences, Cheeloo College of Medicine, Shandong University, 44 West Wenhua Road, Jinan, Shandong 250012, P.R. China

^2^ Laboratory of Materials, Macromolecules, and Composites (LaMMAC), Federal University of Technology - Paraná (UTFPR), Apucarana, PR 86812-460, Brazil.

^3^ Group of Polymers and Composite Materials (GMPC), Department of Chemistry, State University of Maringá (UEM), Maringá, PR 87020-900, Brazil.

^4^ Department of Chemical and Biological Engineering, Colorado State University (CSU), Fort Collins 80523, USA.

^5^ Key Laboratory of Molecular Pharmacology and Drug Evaluation, Yantai University, Yantai, P.R. China

^6^ Key University Laboratory of Pharmaceutics & Drug Delivery Systems of Shandong Province, School of Pharmaceutical Sciences, Cheeloo College of Medicine, Shandong University, 44 West Wenhua Road, Jinan, Shandong 250012, P.R. China

^7^ Pediatric Pharmaceutical Engineering Laboratory of Shandong Province, Shandong Dyne Marine Biopharmaceutical Company Limited, Rongcheng, Shandong 264300, P.R. China

^8^ Chemical Immunopharmaceutical Engineering Laboratory of Shandong Province, Shandong Xili Pharmaceutical Company Limited, Heze, Shandong 274300, P.R. China

*** Correspondence:**Prof. Zhongxi Zhao
[zxzhao@sdu.edu.cn](mailto:zxzhao@sdu.edu.cn)

Supporting Figures


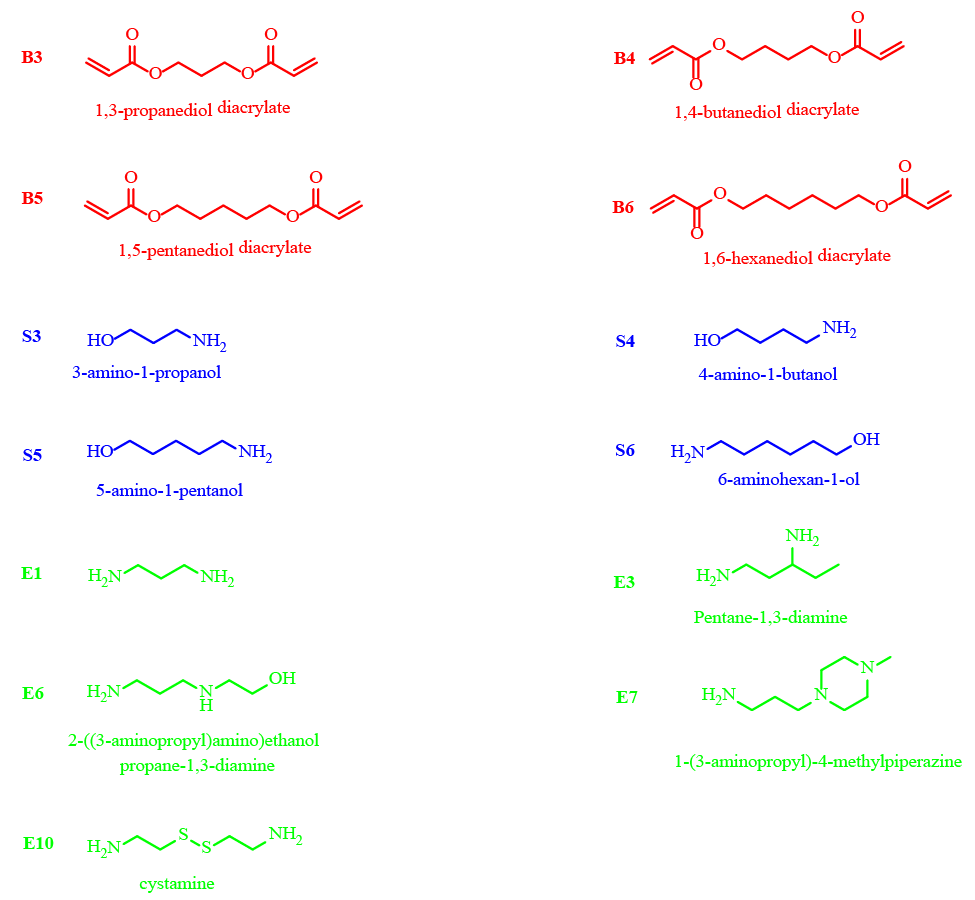


**Figure S1.** Monomers list that is used to synthesis a vast library of PβAEs


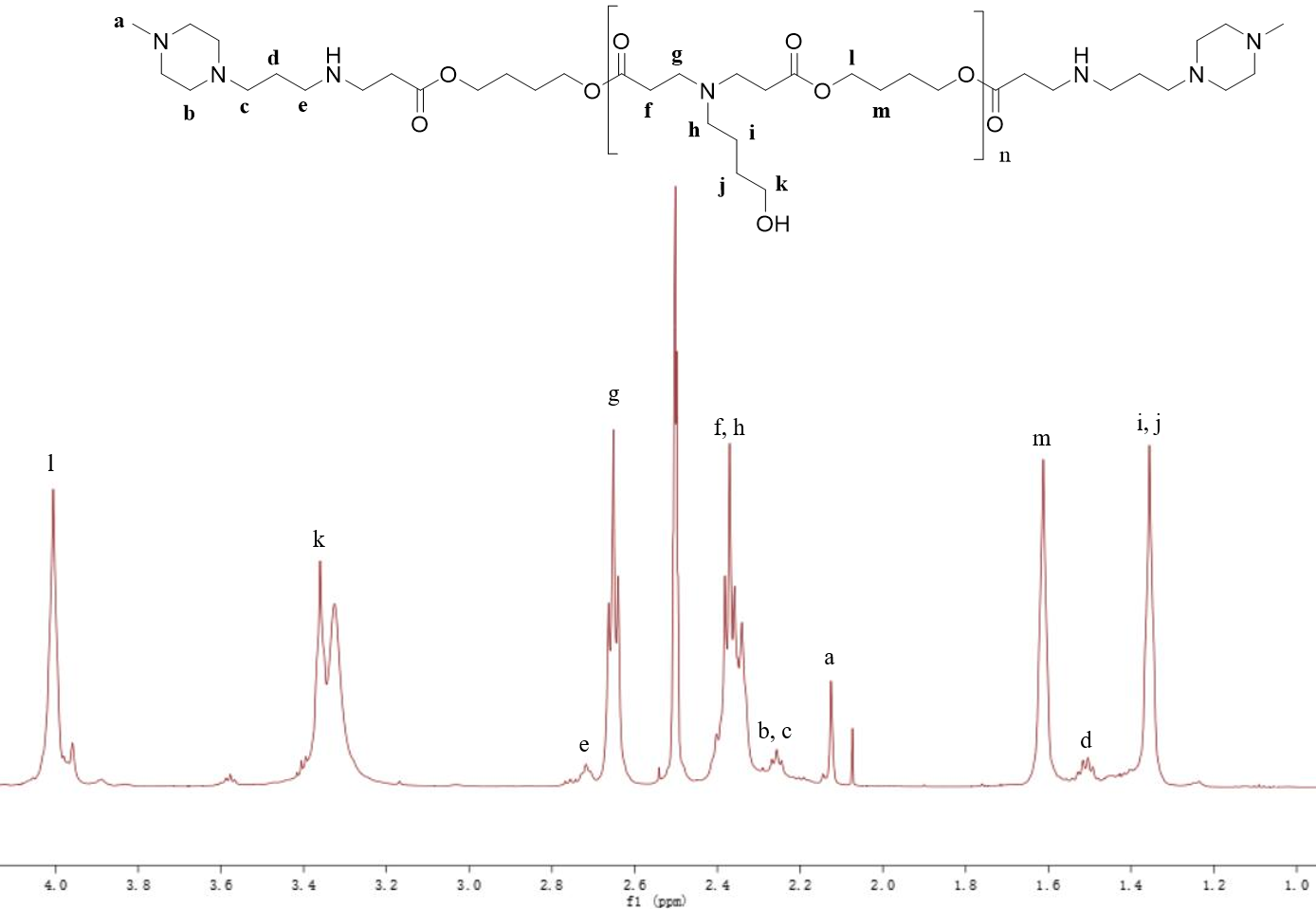


1.47-1.29 (m, NCH_2_C***H_2_***CH_2_CH_2_OH and NCH_2_CH_2_C***H_2_***CH_2_OH)

1.55-1.47 (t, NCH_2_C***H_2_***CH_2_N<(CH_2_CH_2_)_2_>NCH_3_)

1.67-1.55 (m, COOCH_2_C***H_2_***C***H_2_***CH_2_OOC)

2.13(s, NCH_2_CH_2_CH_2_N <(CH_2_CH_2_)_2_>NC***H_3_***)

2.43-2.21 (t, COOC***H_2_***CH_2_NCH_2_C***H_2_***OOC and t, NC***H_2_***CH_2_CH_2_CH_2_OH and m, NC***H_2_***CH_2_C***H_2_***N<(C***H_2_***C***H_2_***)_2_>NCH_3_)

2.69-2.58 (t, COOCH_2_C***H_2_***NC***H_2_***CH_2_OOC)

3.42-3.34 (m, NCH_2_CH_2_CH_2_C***H_2_***OH)

4.05-3.97 (m, COOC***H_2_***CH_2_CH_2_C***H_2_***OOC)

**Figure S2.** ^1^H NMR spectroscopy of PβAE-447


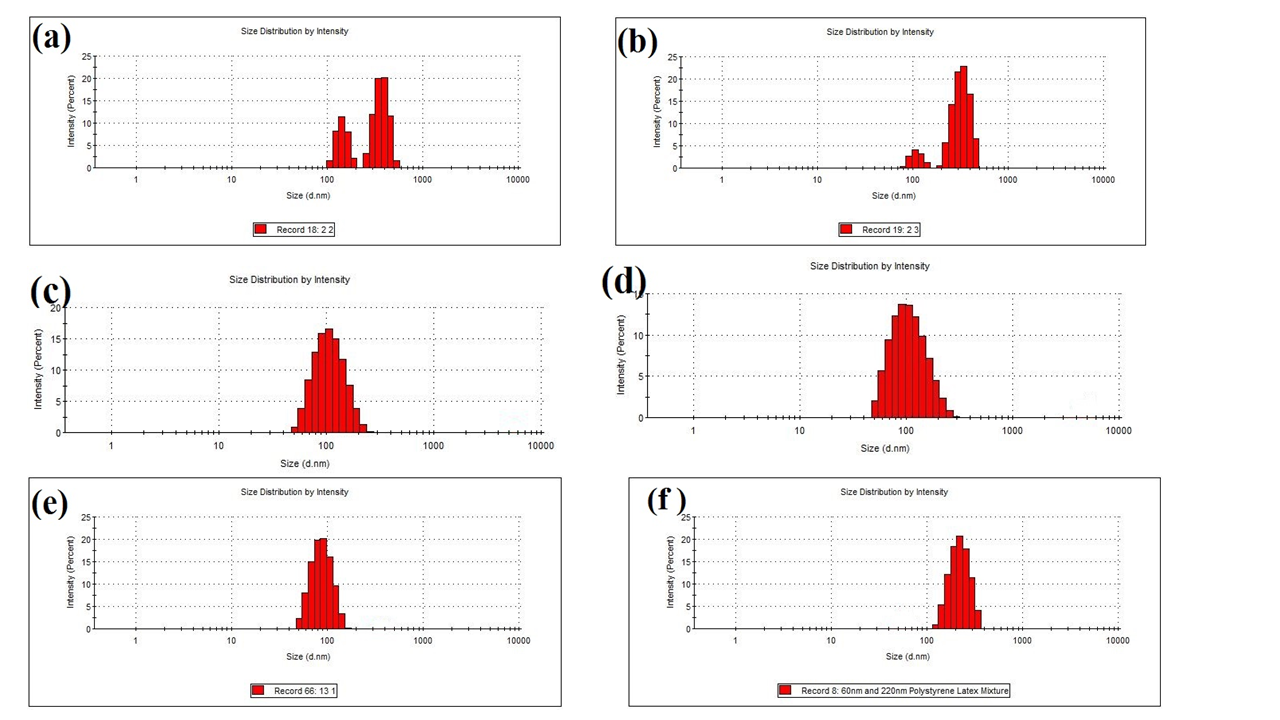


Figure S3. Characterization studies of PβAE-447/pDNA nanoparticles Size: a) as-prepared PβAE-447/pDNA nanoparticles in FBS after four hours at room temperature: b) as-prepared PβAE-447/pDNA nanoparticles in NaCl after four hours at room temperature: c) PβAE-447/pDNALypholized nanoparticles: d) as-prepared PβAE-447/pDNA nanoparticles: e) PEGylated PβAE-447/pDNA nanoparticles in FBS: f) PEGylated PβAE-447/pDNA nanoparticles in NaCl


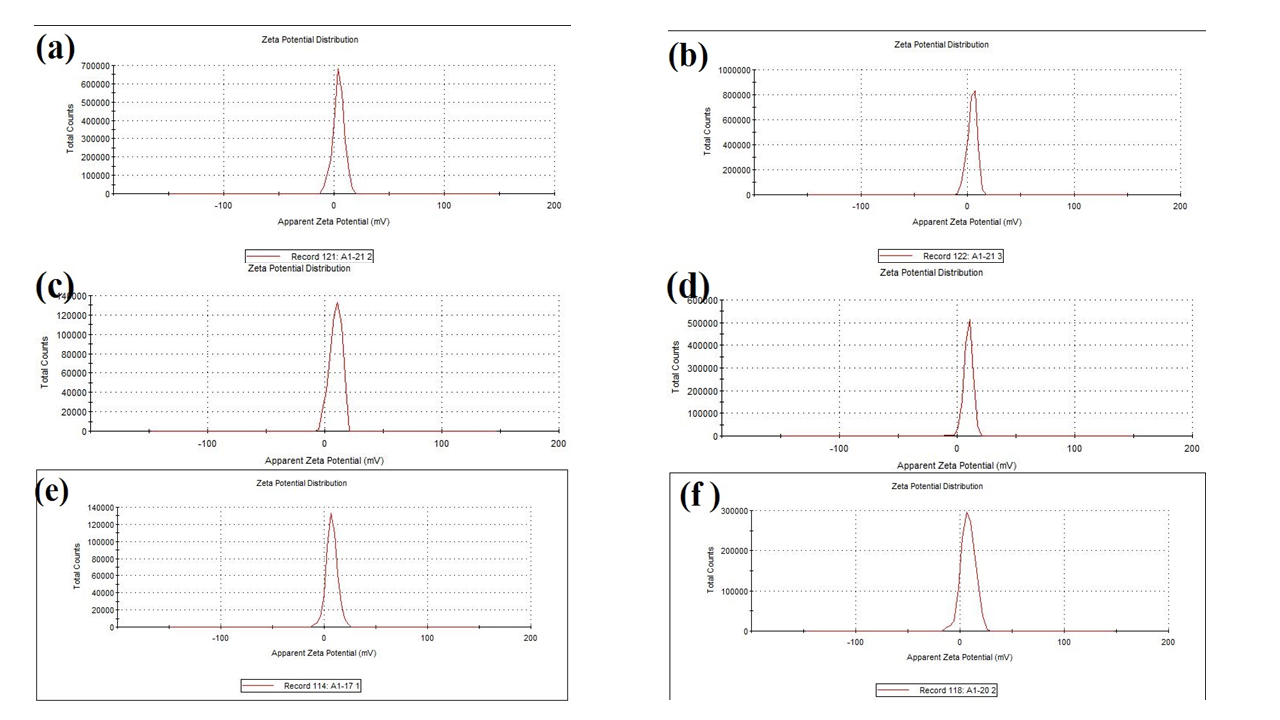


Figure S4. Characterization studies of PβAE-447/pDNA nanoparticles Zeta potential: a) as-prepared PβAE-447/pDNA nanoparticles in FBS after four hours at room temperature: b) as-prepared PβAE-447/pDNA nanoparticles in NaCl after four hours at room temperature: c) PβAE-447/pDNA Lypholized nanoparticles: d) as-prepared PβAE-447/pDNA nanoparticles: e) PEGylated PβAE-447/pDNA nanoparticles
